# The olivine-ringwoodite transformation triggers deep slab seismicity and rheological weakening

**DOI:** 10.1038/s41467-026-71661-z

**Published:** 2026-04-16

**Authors:** Rikuto Honda, Tomoaki Kubo, Masaaki Miyahara, Takuya Iwasato, Yuichiro Mori, Yuji Higo, Yumiko Tsubokawa, Yuta Goto, Akio Suzuki, Yuki Shibazaki

**Affiliations:** 1 Department of Earth and Planetary Sciences, Graduate School of Sciences, Kyushu University, Fukuoka, Japan; 2 Department of Earth and Planetary Sciences, Faculty of Sciences, Kyushu University, Fukuoka, Japan; 3 Graduate School of Advanced Science and Engineering, Hiroshima University, Higashi-Hiroshima, Japan; 4 Shin-Nippon Nondestructive Inspection Co. Ltd., Kitakyushu, Japan; 5 Department of Earth and Planetary Science, Graduate School of Science, The University of Tokyo, Hongo, Tokyo Japan; 6 Japan Synchrotron Radiation Research Institute, Hyogo, Japan; 7 Department of Earth Science, Graduate School of Science, Tohoku University, Sendai, Japan; 8 Institute of Materials Structure Science, High Energy Accelerator Research Organization (KEK), Tsukuba, Japan; 9 Present Address: Institute for Geothermal Sciences, Graduate School of Science, Kyoto University, Beppu, Japan

**Keywords:** Geodynamics, Seismology

## Abstract

The subducting cold oceanic plates (slabs) exhibit two paradoxical deformation behaviors: deep seismicity and rheological weakening within the mantle transition zone (MTZ, ~400–700 km depths). Although the transformation of metastable olivine wedge (MOW)^1,2^ in cold slabs has been proposed as a possible trigger for both behaviors^3–10^, direct experimental evidence remains limited to understand the processes linking them. Here we report experimental results on the transformation-deformation coupling at MTZ pressures (~20 GPa). Ringwoodite is produced as nano-polycrystalline lamellae (NPL) under uniaxial stress. Thin NPL trigger unstable slips with coseismic stress drops by grain-size sensitive creep coupled with thermal instability at ~760–860 °C. Thickening of NPL at ~950–1,330 °C stabilizes the deformation with enhancing the transformation utilizing their incoherent nature. Thus, the formation of NPL and their grain-size sensitive creep play key roles in temperature-dependent transformation-deformation coupling, which explains both deep seismicity near the MOW and rheological weakening outside the MOW.

## Abstract (web summary)

Deformation experiments on olivine at mantle transition zone pressures (~20 GPa) demonstrate temperature-dependent transformation–deformation coupling that explains both deep-focus earthquakes and deep slab weakening.

## Introduction

Deep-focus earthquakes occur in cold MTZ slabs at depths down to ~680 km, and the occurrence rate reaches a maximum at ~600 km depth, corresponding to a pressure of ~21 GPa^11^. It has been a long-standing problem why and how the faulting occurs in silicate rocks far beyond the brittle-plastic transition (BPT) pressure (~2 GPa)^3,4^. Experimental demonstration of shear localization and shear instability at MTZ pressures is necessary to understand the detailed processes. On the other hand, seismic tomography studies^12^ showed extensive deformation and stagnation of cold subducting slabs at the bottom of the transition zone. This phenomenon requires not only resistance to subduction, such as the negative Clapeyron slope associated with the post-spinel transformation and metastable transformations, but also the weakening of the slab in spite of the low temperature of this region^13^. This cannot be simply interpreted by strongly temperature-dependent viscosity in rocks commonly assumed. These two rheological features of cold slabs are both apparent paradoxes of rock mechanics in the plastic regime. The interaction between transformations and deformation is a key to solving these issues because most constituent minerals in subducting slabs undergo high-pressure transformations at MTZ pressures.

The grain-size reduction due to the metastable olivine transformation can be a possible trigger for both deformation behaviors. For example, previous studies have demonstrated that the olivine transformation causes shear instability by forming anticracks or nanoshear bands (NSBs) filled with fine-grained spinel phase^5–7^. However, most deformation experiments were indirect, which performed at ~2–5 GPa near the BPT pressures accompanied by the analogue olivine transformations. Most recently, a different model was advocated for the shear instability induced by the olivine-wadsleyite (modified spinel structure) transformation at ~13–15.5 GPa, where it was caused by connecting lenticular packets filled with nanocrystalline olivine and wadsleyite^8^. In this way, although several interesting processes regarding the grain-size reduction have been proposed for the origin of the deep earthquakes, it is still questionable whether they really work at higher pressures corresponding to the bottom of MTZ where ringwoodite becomes stable and the rate of deep seismicity increases. Additionally, previous studies have also shown that the transformed fine-grained ringwoodite exhibits grain-size sensitive creep and suggested it as an important process for the rheological weakening of MTZ slabs^9,10^. Thus, the grain-size reduction of the transformation is a key process for the two contrasting deformation behaviors of cold MTZ slabs. However, it is still poorly understood what processes link the shear instability and rheological weakening.

In this work, to address these issues, we conducted deformation experiments of mantle olivine under the conditions for the bottom of MTZ using the D-111 type deformation apparatus (Supplementary Figs. 1–4). The coupling behaviors between transformation and deformation were investigated by in-situ synchrotron X-ray observations (Supplementary Figs. 5–7) and acoustic emission (AE) measurements (Supplementary Figs. 8, 9) together with microstructural observations of recovered samples. The starting material of polycrystalline San Carlos olivine was compressed to ~20 GPa at room temperature, and then uniaxially deformed at ~570–1330 °C with a constant anvil displacement rate. A total of nine runs were conducted in both quenching and in-situ X-ray observation methods, including seven runs with AE measurements (Supplementary Table 1). The other details of experimental procedures are given in Methods. These investigations reveal that the transformation of metastable olivine to ringwoodite under differential stress plays a critically important role in triggering both shear instability and rheological weakening.

## Results and discussion

### Homogeneous deformation at high T

**Fig. 1:**
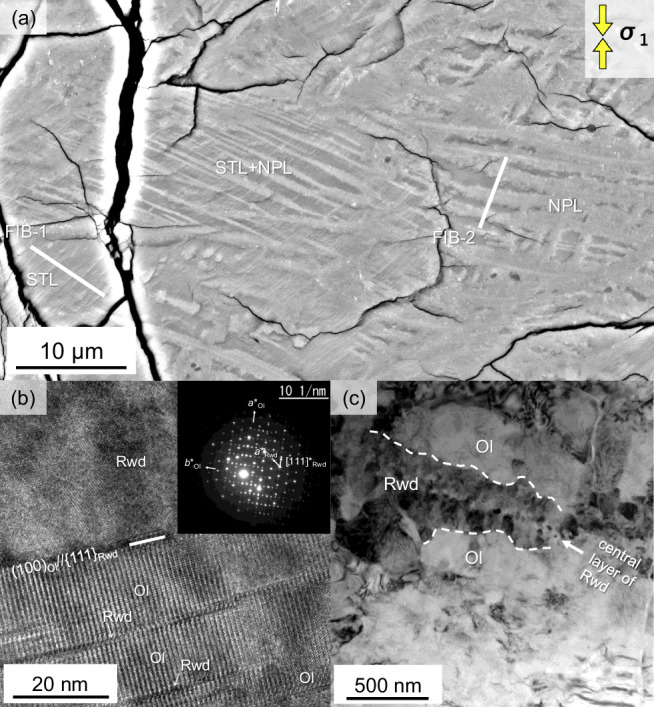
Microstructures of samples recovered from high temperatures. Homogeneous deformation with extensive transformation was observed in a temperature ramping path to 1330 °C (Run oldt12). The vertical direction corresponds to the orientation of the maximum principal stress axis σ_1_. **a** Backscattered electron image showing a development of two types of ringwoodite lamellae of single crystalline topotactic lamellae (STL) and nano-polycrystalline lamella (NPL). Transmission electron microscopy images of STL (**b**) and NPL (**c**), obtained from regions excavated using a focused ion beam (FIB) at locations FIB-1 and FIB-2 in (**a**), respectively. The inset in (**b**) is a selected area electron diffraction pattern between olivine (Ol) and ringwoodite (Rwd) showing topotactic relations between these phases. A thick NPL in (**c**) shows a layered structure consisting of a fine-grained central layer and the outer layers formed by secondary nucleation.

We observed systematic changes in the transformation-deformation coupling behaviors with temperatures. The transformation largely proceeded more than ~80% at higher temperature than 1070 °C (Runs oldt12 and L64), where two types of ringwoodite lamellae were produced in olivine grains (Fig. 1a). One is single-crystalline topotactic lamellae (STL) with a crystallographic orientation relationship of (100)_Ol_ // {111}_Rwd_ (FIB-1 in Fig. 1a, b). This type of lamellae had also been observed at large overpressures even when the sample was not deformed^14^. Another one is nano-polycrystalline lamellae (NPL) with random orientations (FIB-2 in Fig. 1a, c). NPL in this run had multi-layered textures, in which nanograins of ringwoodite with a size of ~30 nm formed the central layer, and the outer layers developed on each side by secondary nucleation and growth of elongated and roughly oriented grains with ~100–200 nm in length perpendicular to the central layer. These two types of lamellar textures had also been observed in L6 shocked chondrites^15,16^. The transformation originated from NPL predominantly proceeds compared to those from STL both in experiments and meteorites. NPL tended to lie at high angles to the deformational axis, while STL developed randomly in our experiments (Supplementary Fig. 10). In this temperature range, homogeneous deformation was developed associated with the bulk transformation, and both NPL and STL did not cause localized deformation.

### Formation of nanoshear bands at medium T

**Fig. 2:**
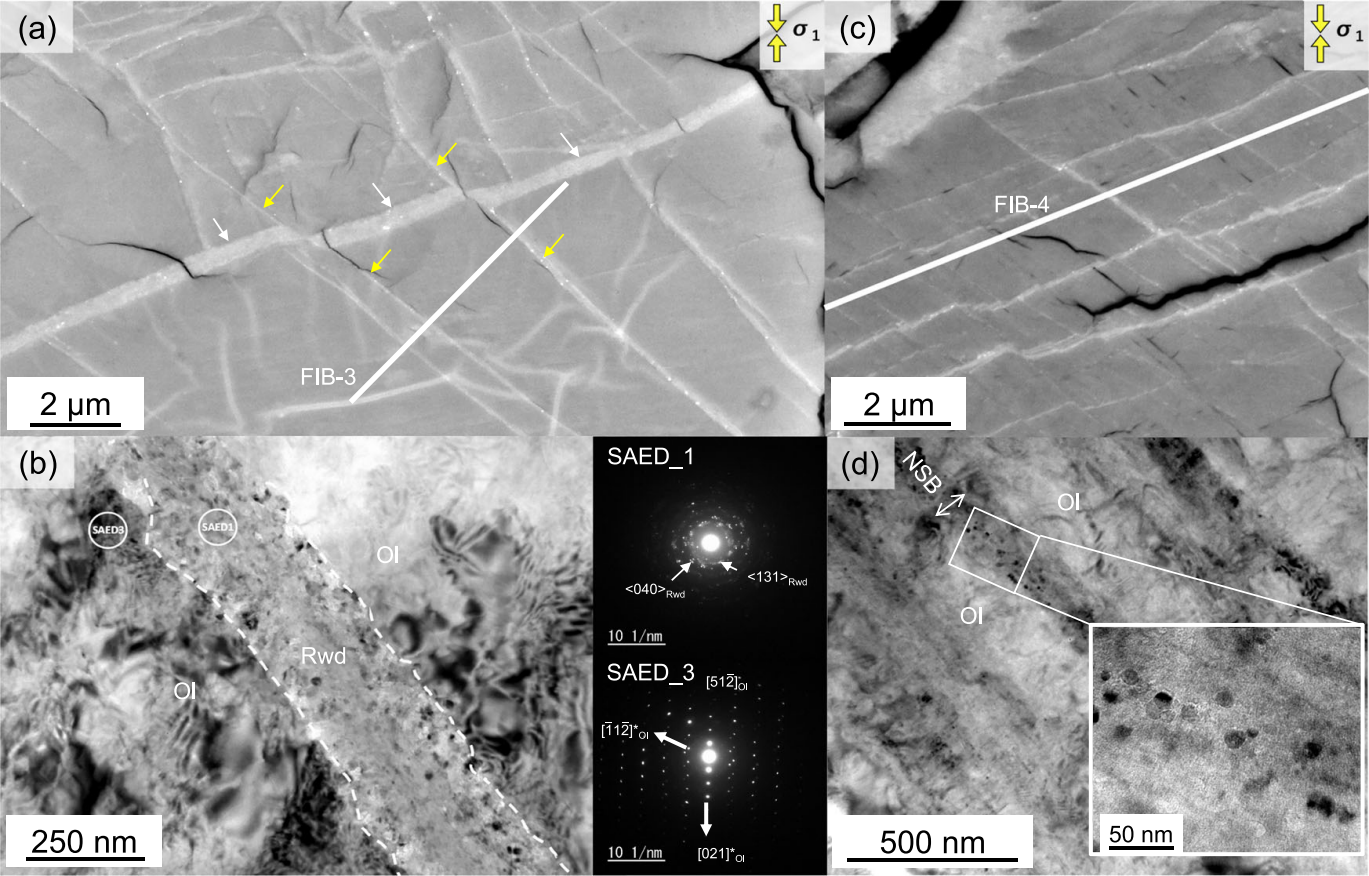
Microstructures of samples recovered from medium and low temperatures. Shear localization associated with the formation of nano-polycrystalline lamella (NPL) (nanoshear bands, NSBs) was observed at 1020 °C (**a**, **b**, Run oldt23). The thin foils used for transmission electron microscopy (TEM) observation were extracted by a focused ion beam (FIB). **a** Backscattered electron image (BEI) showing the broad NPL (white arrows) sheared by the sharper ones (yellow arrows) with slip displacement of ~0.6 μm. **b** TEM image and selected area electron diffraction (SAED) patterns (insets) from FIB-3 in (**a**) revealed the NSBs consist of randomly-oriented fine-grained ringwoodite (Rwd) with a grain size of ~20–30 nm. The shear localization became unstable accompanying with acoustic emissions at lower temperatures of 830° C (**c**, **d**, Run L78). **c** BEI showing thinner NSBs with ~100 nm thickness developed in a conjugated form. **d** TEM image from FIB-4 in (**c**) showing that an NSB consists of finer-grained ringwoodite, where the magnified inset shows the smallest grain size of ~10 nm. The parental olivine (Ol) grains deformed at medium and low temperatures are heavily strained, although clear TEM imaging was difficult due to the relatively thick FIB foil and the high density of tangled dislocations (Fig. 2b, d).

Similar transformation textures but with shear localization were observed at the lower temperatures of 950–1020 °C (Runs oldt23 and L76). At 1020 °C (Run oldt23), the NPL became narrow ~0.3–1 μm in width, where the sharp NPL cut the broader one with slip displacements of ~0.6 μm, forming nanoshear bands (NSBs, Fig. 2a). Although STL were also observed, they did not contribute to localized deformation. TEM observations revealed that the sharp NSBs with slip displacements consisted of randomly-oriented nanograins of ringwoodite with a size of 20–30 nm (Fig. 2b), which were thought to be identical to the central layer of the broader NPL observed in Run oldt12 (Fig. 1c). The parental olivine was heavily deformed with numerous tangled dislocations. We did not observe clear evidence of melting such as the presence of amorphous phases and dendritic grains. In order to know whether the reaction-induced shear localization was unstable triggering shear instability or not, we carried out the additional quenching experiments with AE measurement at 950 °C (Run L76) under similar conditions as Run oldt23. However, no AEs were detected although similar NSBs were present in the recovered sample, suggesting that the sliding along NSBs was stable at these temperatures.

**Fig. 3:**
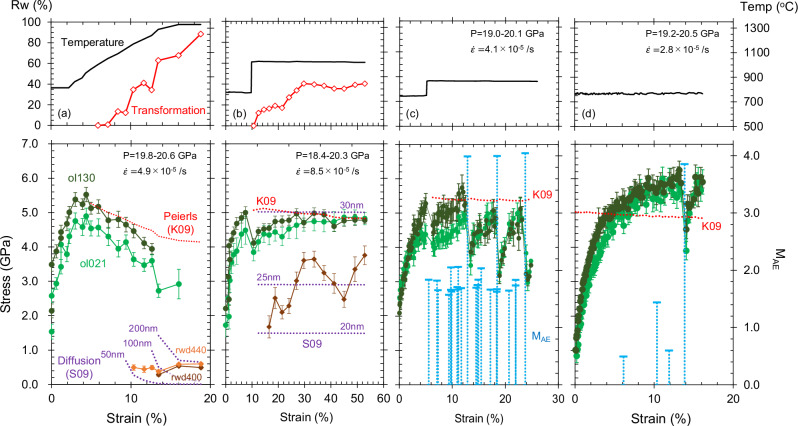
Stress-strain curves, transformation rates, and acoustic-emission activities during the deformation stage at various temperatures. Temperature (black) and transformed fraction (red) are shown in the upper panels, and magnitude of acoustic emissions (M_AE_) is shown as blue dotted bars in the lower panels. Flow strength of newly appeared ringwoodite (rwd) derived from (400) and (440) reflections (brown and orange diamonds) is lower than that of parental olivine (ol) derived from (130) and (021) reflections (dark and light green circles) at high (**a**: Run oldt12 in the temperature ramping path to 1330 °C) and medium temperatures (**b**: Run oldt23 at 1020 °C), which are reasonably interpreted by flow laws of grain-size sensitive diffusion creep^17^ (purple dotted line, S09) and the low-temperature plasticity^19^ (red dotted line, K09), respectively. Unstable behaviors such as stress drops and acoustic-emission activities were detected in the lower temperature runs (**c**: Run oldt62 at 860 °C, **d**: Run oldt51 at 760 °C), where the amount of ringwoodite is very few and cannot be observed by X-ray diffraction. Uncertainties of stress are shown as error bars. Pressure (*P*) and strain rate ($\dot{\varepsilon }$) are also shown.

The stress-strain curves obtained in these runs indicated that the newly appeared ringwoodite grains are much weaker than the parental olivine (Fig. 3a, b). The olivine stress gradually decreased from ~5–6 GPa to ~3 GPa in the temperature ramping run (Run oldt12, Fig. 3a), and was almost constant during the isothermal deformation (Run oldt23, Fig. 3b). In contrast, the stress of ringwoodite remained low ~0.5 GPa with increasing temperature (Fig. 3a) and exhibited hardening at constant temperature (Fig. 3b). These creep behaviors in ringwoodite can be reasonably interpreted as grain-size sensitive creep of NPL associated with grain growth. We calculated flow stress in the diffusion creep regime based on the Si diffusivity in ringwoodite^17^, as shown by the dotted lines in Fig. 3a, b, which vary depending on grain size. The seemingly unusual behaviors—constant flow stress with increasing temperature (Fig. 3a, Run oldt12) and hardening at a constant temperature (Fig. 3b, Run oldt23)—can be approximately explained by the increase in grain size due to grain growth. Although a quantitative discussion is challenging due to relatively large uncertainty and variability in stress values, resulting from low diffraction intensity caused by the low fraction transformed and its heterogeneous distribution within the sample, these grain sizes are generally consistent with the microstructural observations from each run and roughly follow the grain growth kinetics^18^. The appearance of weaker fine-grained ringwoodite and its hardening during isothermal deformation have also been reported in the previous study^10^ although the transformation and deformation microstructures were unclear. Our results directly demonstrated that the stress-induced formation of NPL is a key for weakening via grain-size sensitive creep, providing evidence that fills the gap in the limitations of their analysis.

In comparison, the high flow stress, its small temperature dependence (Fig. 3), and heavily strained texture (Fig. 2b) in olivine suggest that the low-temperature plasticity is dominant for the flow of the parental phase. The olivine strength can be largely explained by the pressure dependent flow law of the low-temperature plasticity with an activation volume of ~30 cm^3^/mol^19^. It is plausible that the large contrast in strength between the parental olivine and the newly appeared ringwoodite of NPL causes shear localization, resulting in the formation of NSBs.

### NSBs become unstable at low T

At the lower temperature of 760–860 °C, the sliding along NSBs becomes unstable as evidenced by AE activities and coseismic stress drop (Fig. 3c, d). We detected 4–43 AE events with 28–32 mV trigger levels in four isothermal deformation runs (Supplementary Fig. 9). Most of the AEs were located within the sample region considering the effects of the lack of several transducers (Supplementary Fig. 8). The stress of olivine suddenly dropped by ~1.5 GPa associated with large AEs after reaching the maximum value of ~6 GPa, and soon recovered to the original level by the additional ~5% strains. This behavior repeated several times at 860 °C (Run oldt62, Fig. 3c) while it occurred once at 760 °C (Run oldt51, Fig. 3d). Some stress drops accompanied with an increase in strain by ~2% corresponding to the axial displacement of ~10 μm. Although ringwoodite was not detected in these runs by in-situ XRD measurements, we found that the similar but thinner and a greater number of NSBs were distributed in parental olivine grains (Fig. 2c). They consisted of finer-grained ringwoodite with a grain size of ~10 nm (Fig. 2d) and are oriented at an angle of 40–50° to the deformation axis (Supplementary Fig. 10). These results indicate that the transformation-induced shear localization becomes unstable fault slip in this limited temperature range, triggering shear instability and AEs.

We also found open fractures inclined at ~45–50° to the deformation axis, going through the recovered specimens (Supplementary Fig. 11). This may suggest that, in addition to microscopic NSBs, macroscopic failure (the fault going through the specimen) also occurs accompanying AEs and the stress drop. However, the blowout of the sample assembly and stick slip of the split-hexagonal piston sometimes occurred on release of pressure at room temperature, which makes it difficult to properly interpret microstructures of this region.

The relation between a stress drop (Δ*σ*) and a slip displacement (*D*) is described as Δ*σ*=*αGD*/*L*, where *α* is a geometric constant of order unity, *G* is the shear modulus (~100 GPa)^20^, and *L* is the length of the slip zone^6,21^. The stress drop of ~1.5 GPa may be explained by considering the observed slip displacement (*D* ~ 300 nm, Fig. 2c) and the length of the slip zone (*L* ~ 20 µm, comparable to the olivine grain size) in NSBs. However, this means that slip displacements along microscopic NSBs simultaneously form in all of the olivine grains, which is unlikely. The NSBs in certain parts of the olivine polycrystals may be related to the undetectable stress drops associated with smaller AEs. On the other hand, if we consider the macroscopic failure as mentioned above, the stress drop can be reasonably explained by taking *D* ~ 15 µm and *L* ~ 1 mm. This is a plausible explanation as the source of the stress drop associated with large AEs although it is difficult to directly measure the *D* value due to the fracturing when recovering the sample. The undetectable stress drops associated with smaller AEs can also be explained by the smaller coseismic slip in this macro-scale faulting.

The amount of ringwoodite is very few at seismogenic temperatures of 760–860 °C. The localized deformation bands developed in the parental olivine, and ringwoodite was infrequently found along them (Supplementary Fig. 12). TEM observations suggest that the deformation bands were formed as a result of dislocation pileups in the olivine grain with high dislocation densities, which are characteristic microstructures deformed at low temperatures^22^. These deformation features in olivine were also observed in the recovered sample from the lowest temperature run at 570 °C (Run L125), in which no AEs were detected and no ringwoodite was found. Thus, the formation of the deformation bands in olivine itself does not cause shear instability but would provide the intracrystalline incoherent nucleation site as a precursor for NPL. Nucleation of nano-grained ringwoodite on the planar defects results in the formation of weak planes in the parental olivine, leading to shear localization and shear instability.

### Transformation-induced thermal instability

It has been suggested that polymorphisms, significant volume changes, and high latent heats were important three factors causing transformation faulting^4^. The olivine-spinel transformations in both germanate and magnesium silicate meet these requirements, and the characteristics of the unstable NSBs observed at ~20 GPa in mantle olivine (Fig. 2c) show some similarities to those observed at lower pressures of ~1–5 GPa in germanate olivine^6,7,23^. They are very thin (less than ~100 nm thickness) and consist of very fine grains (a few tens of nm in size) without melting. These micro faults are almost oriented around ~45° to the σ_1_ direction, which is larger than those observed in brittle failure involving frictional sliding. These similarities imply that the instability mechanism induced by the formation of NSBs is ubiquitous, extending well beyond the BPT pressure.

In addition to these findings, we obtained the experimental evidence of transformation-induced stick-slip behavior (repetitive stress drops accompanied by transient slip events), providing an important insight on the similarity between shallow and deep earthquakes. The stress drop larger than 1 GPa repeatedly occurs associated with AEs (Fig. 3c), which was unclear in the previous lower-pressure experiment^6^. The strength of parental olivine is much higher at mantle transition zone pressures and the release of latent heat is more significant in magnesium silicate^4^, which likely contributes to the occurrence of the stick slip instability at higher pressures.

In the previous studies on the germanate olivine, the effective frictional coefficients *μ* for unstable NSBs have been indirectly estimated to be less than ~0.1–0.01 considering the microstructural observations and the amount of shear heating^6,24^. We directly measured the large contrast in strength between olivine and ringwoodite during the localized deformation without AEs (Run oldt 23, Fig. 3b), which gives *μ* of ~0.04–0.09 for stable NSBs at medium temperatures (Methods). For the lower temperature runs involving unstable NSBs with AEs (Runs oldt 51 and 62), we estimated the diffusion creep strength of ringwoodite derived from Si diffusivity^17^, as demonstrated in runs oldt12 and 23 (Fig. 3a, b), because we were unable to measure the strength directly via X-ray diffraction due to the low transformed fraction. The result showed that the strength of ringwoodite is larger than that of olivine in those runs, suggesting that the shear localization would not occur. However, this calculation may not accurately reflect the sliding conditions at the precise moment of NPL nucleation. In order to capture the features of the transformation-induced instability, we recalculated the instantaneous friction coefficients considering the initial grain size of ringwoodite and adiabatic increase of the local temperature (~240–270 °C) due to latent heat release of the transformation (Methods). As a result, the release of latent heat effectively reduces the instantaneous friction coefficient to around 10^-3^–10^-5^, and the NPL weakens sufficiently to cause shear localization, even at the low temperatures where the unstable slip has been observed.

The low-friction resistance alone does not necessarily lead to shear instability, as solid flow primarily exhibits velocity-strengthening behavior (i.e., positive relationship between strain rate and stress)^21^. In fact, stable slip was observed at higher temperatures of 950–1020 °C. An additional key process for triggering instability is thought to be frictional heating, where shear localization induced by latent heat release generates further heat through friction. Our experiments demonstrated that this velocity-weakening instability occurs adiabatically, prior to the dissipation of the local temperature rise caused by latent heat release through heat conduction on the microsecond timescale (Methods).

**Fig. 4:**
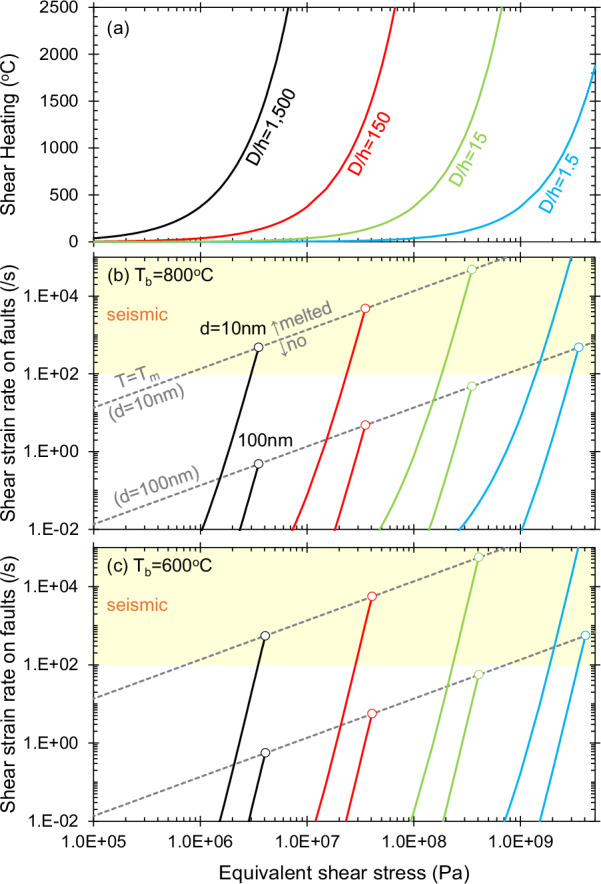
Shear heating and the resultant shear strain rate on faults. **a** Temperature increases by shear heating for various *D/h* values as a function of shear stress. The resulting shear strain rates on faults are calculated based on the diffusion creep in ringwoodite considering temperature increases caused by the latent heat release and the shear heating, at background temperatures *T*_*b*_ representative of laboratory conditions (**b**, 800 °C) and slab conditions (**c**, 600 °C). Grain sizes in the range *d* ~ 10 to 100 nm are also considered in these calculations. The localized deformation of nano-grained ringwoodite can achieve seismic strain rates (i.e., >10^2^ s^−1^, yellow hatched zone) or cause frictional melting (above melting temperature *T*_*m*_: the gray dashed lines)^62^ both in lab (larger shear stress and smaller *D/h* value) and slab (smaller stress and large *D/h* value) scales.

Frictional heating depends on sliding conditions such as slip distance *D*, the thickness of the fault plane *h*, and the shear stress on the fault (Methods). We estimated the temperature increases by the frictional heating and the resultant shear strain rates on the fault plane in Fig. 4. The frictional heating along the grain-scale unstable NSBs observed in our experiments (*D*/*h* ~ 1.5 under the equivalent shear stress of ~2–2.5 GPa) is expected to increase the temperature by ~750–940 °C during slips (Fig. 4a) in addition to the latent heat effect. This enables the slip in the seismic strain rates (e.g., >~10^2^ s^-1^) by the diffusion creep of NSBs without melting, when we consider the grain size of ~10 nm and the background temperatures of ~800 °C (Fig. 4b). Although the precise sliding conditions for the possible macro-scale faulting in this study are not clear, the *D*/*h* value is expected to be larger than that of the grain-scale NSBs, as observed in previous analogue studies^6,7,24^. The larger *D*/*h* value gives the larger amount of shear heating, leading to unstable slip and even to localized melting.

We observed unstable slip within a limited temperature range of 760–860 °C. The lower limit corresponds to the initiation of NPL nucleation, which is necessary for shear localization. The upper limit may be controlled by the shear stress on the fault and, consequently, the amount of frictional heat. At temperatures above the upper limit, ringwoodite is initially produced with a lower strength than the parental olivine, even without considering the latent heat effect (e.g., Run oldt 23). In this case, although shear localization occurs, frictional heating is likely insufficient due to the low shear stress on the fault, thereby inhibiting instability. Thus, stress-induced NPL formation and grain-size sensitive creep, coupled with latent heat release and shear heating, are essential processes responsible for the laboratory earthquakes observed within the limited low-temperature range.

### Deep slab seismicity and rheological weakening

Previous studies have shown that deep earthquakes occur within a limited depth range and are highly sensitive to temperature, with their occurrence rate peaking at around 600 km depth^11^. In Fig. 4, we explore whether transformation-induced thermal instability can explain the origin of deep earthquakes on the larger spatial and temporal scales of subducting slabs, considering variations in temperature, differential stress, grain size, and *D*/*h* values relative to our laboratory experiments.

A previous study suggested that the locations of deep-focus earthquakes are stringently restricted by temperatures below ~750 °C in the Tonga slab^25^. The temperature conditions for the shear instability in our experiments (~760–860 °C) are slightly higher than this threshold. However, the formation of NPL, which marks the initial step of instability, is kinetically controlled and could occur at lower temperatures over the longer timescales of subducting slabs. For example, the transformation from metastable olivine to ringwoodite has been proposed to occur at 550 °C in the coldest regions of the Mariana slab^2^. Thermal modeling of subducting slabs^2,26,27^ has further indicated that latent heat release from such metastable phase transformations can raise local temperatures by up to ~ 200 °C. During the initial step of this latent heat effect, the weakened NPL may promote shear localization via grain-size sensitive creep, as demonstrated in the NSBs of our experiments.

Stresses in such cold subducting slab, which are cold enough to retain a metastable olivine wedge and generate deep seismicity, have been estimated to reach several GPa, based on both modeling and observational data^28–32^. The flow strength of metastable olivine far beyond the equilibrium boundary becomes high due to the large pressure effect, resulting in the large strength contrast between MOW and the surrounding ringwoodite. This leads to the stress concentration in MOW, which promotes the NPL formation. In addition, further stress concentration is expected in a composite slab subducting alongside MORB^32^. On the other hand, observed stress drop due to deep earthquakes range from several to tens of MPa^31^, providing a minimum estimates of slab stress.

Although the stress range under slab conditions is broad and generally lower than that applied in our experiments, frictional heating depends on both stress and the *D*/ℎ ratio, which are correlated (Fig. 4a) and together control the amount of heat required to achieve seismic strain rates or frictional melting (Fig. 4b, c). In laboratory experiments, the *D*/ℎ ratio is necessarily very small, and instability requires stresses on the order of GPa, as demonstrated here. In contrast, the *D*/*h* values associated with deep earthquakes are thought to be much larger, reaching ~1000 for the 2013 Okhotsk event^33^ and ~4000 for the 1994 Bolivian deep earthquake^34^, such that stress of only several MPa are sufficient.

While frictional heating may promote further grain growth within NSBs, smaller grain sizes weaken NSBs and increase shear strain rates, whereas larger grain sizes harden NSBs and enhance shear heating. Exploring a plausible grain-size range from 10 to 100 nm in Fig. 4 indicates that, despite partial heat loss through dissipation, either seismic strain rates or frictional melting can be achieved for realistic *D*/ℎ values. Thus, transformation-induced shear instability mediated grain-size sensitive creep provides a plausible mechanism for deep earthquakes occurring at depths of ~600 km.

The previous study^10^ demonstrated the grain-size sensitive creep of newly transformed fine-grained ringwoodite, which leads to slab weakening. Our study supports their pioneering work by clarifying the microstructural processes and further provides insights into the connection between deep-focus earthquakes and the slab weakening mechanisms, with the formation of NPL playing another essential role. Although both NPL and STL were produced in our experiments, their roles in transformation and deformation seem to be totally different. The STL have random orientations not related to the σ_1_ direction but depending on the crystallographic orientations of parental olivine, suggesting little contributions to the deformation. In contrast, the NPL are very weak due to grain-size sensitive creep and tend to orient at angles related to the σ_1_ direction, initially forming at ~45°, which lead to the shear localization (formation of NSBs), and progressively evolving to higher angle with the bulk deformation (Supplementary Fig. 10). The thin and unstable NSBs become thick and stable above the seismogenic temperatures and serve as additional nucleation sites for extensive transformation at higher temperatures. Although STL have been originally expected to be an additional nucleation site to enhance the bulk transformation rate^2,27^, the NPL play a greater role. This is owing to the incoherent nature of the inter-phase boundaries in the NPL, which contrasts with the STL having coherent inter-phase boundaries. The enhancement of bulk transformation by NPL also effectively reduces the grain size in the bulk of the sample, resulting in homogeneous weakening by grain-size sensitive creep.

We showed that the stress-induced formation of the ringwoodite NPL and its grain-size sensitive deformation play an essential role in the rheological transition with temperatures from the unstable localized deformation coupled with thermal instability to the extensive homogeneous deformation. This process first triggers deep earthquakes at the boundary of MOW and promotes the subsequent transformation leading to the homogeneous weakening outside the MOW above the seismogenic temperature.

## Methods

### High-pressure deformation experiments using D-111 apparatus

We conducted deformation and transformation experiments at the NE7A beamline of the Photon Factory Advanced Ring (PF-AR) for in-situ X-ray observations (Run “oldt” series) and at Kyushu University for the quenching method (Run “L” series) using D-111 type apparatuses MAX-III^35,36^ and QDES-II, respectively (Supplementary Fig. 1). A Kawai-type multi-anvil assembly is used for the D-111 apparatus. The truncated edge length (TEL) of the second-stage anvils is 3 mm. The sample assembly was composed of (Mg, Co)O octahedral pressure medium, LaCrO_3_ cylindrical heater, and Mo electrodes as shown in Supplementary Fig. 2. The sample was enclosed into the NaCl capsule and sandwiched by dense and porous Al_2_O_3_ pistons for uniaxial deformation. Thin Au foils were put on both ends of the sample as strain markers. Graphite and boron-epoxy rods were packed along the X-ray beam path through the heater, the pressure medium, and the gasket in order to increase X-ray transmissivity.

The starting material was polycrystalline olivine that was prepared by sintering a powder of natural olivine from San Carlos, USA, at ~2–3 GPa and 950 °C for 90 min using Kawai-type multi-anvil apparatus (Orange-3000) in Geodynamics Research Center at Ehime University. An electron back-scattered diffraction (EBSD) analysis of the starting material indicates that the grain size is ~10–100 µm (Supplementary Fig. 3). The water content was estimated to be 283 ± 26 wt. ppm H₂O based on Fourie-transform infrared spectroscopy measurements. The presence of a unimodal absorption band, rather than the characteristic hydroxyl peaks within olivine crystals (3200**–**3600 cm^−1^), likely reflects small amounts of H₂O predominantly located at grain boundaries. Cylindrical samples with a height of ~1 mm and a diameter of ~0.8 mm were cored from the sintered olivine by ultrasonic machining and used for the deformation experiment.

The temperature was measured by a W3%Re-W25%Re thermocouple located in the dense alumina piston (Supplementary Fig. 2). The additional experiments using two sets of thermocouples indicated that the temperature at the center of the sample capsule is higher by ~27% ± 10% than that at the thermocouple position, which was considered to estimate the sample temperature from the thermocouple. The pressure was calculated from the unit cell volume of NaCl^37^ for in-situ X-ray observations. In the case of the quenching method, the pressure was calibrated as a function of oil pressure on the basis of the olivine-wadsleyite^38^, the wadsleyite-ringwoodite^39^, and the post-spinel transitions^40^ in Mg_2_SiO_4_ at 1500 °C.

The sample was first compressed to ~20 GPa at room temperature, and then initially deformed at 740–810 °C in most of runs (Supplementary Table 1, Supplementary Fig. 4). After reaching the anvil displacement of ~300 μm (sample strain of ~2–8%), we started to increase the temperature at a constant rate of 5.5 °C/min for the temperature ramping experiment (Run oldt12), or quickly changed the temperature to the target values for the isothermal experiments. In the Runs L125 and oldt51, the sample was deformed at the constant temperature of 570 °C and 760 °C, respectively, without the pre-deformation stage. The deformation was conducted with a constant anvil displacement rate of 300 μm/h in all runs.

### In-situ X-ray observation and data analysis

We obtained stress-strain curves of both parental and newly appeared phases, and transformation rates from in-situ X-ray observations (Fig. 3). For this purpose, monochromatic X-ray with the energy of 60 keV was used to obtain radiography images of the sample (Supplementary Fig. 5) and two-dimensional X-ray diffraction (2D-XRD) patterns (Supplementary Fig. 6a). For acquiring 2D-XRD patterns, one of the second stage anvils at the downstream side was replaced by the sintered diamond anvil bonded with SiC or the WC anvil that is conically slotted by 6° and filled with epoxy. The axial strain *ε* of the sample was estimated from the relation *ε* = -ln(*l*/*l*_0_), where *l* and *l*_0_ are the sample length during and at the beginning of the deformation, respectively. The sample length was measured from the distance between the Au strain markers in radiography images (Supplementary Fig. 5).

Supplementary Fig. 6a shows an example of a 2D-XRD pattern obtained during deformation and transformation. One-dimensional X-ray diffraction (1D-XRD) pattern was obtained by the integration of the 2D-XRD pattern (Supplementary Fig. 6b). The transformed fraction was estimated based on the integrated intensity *I* of diffraction line 021 in olivine relative to the intensity before the transformation in the 1D-XRD patterns. Because the sample volume along the X-ray beam path increased with the axial strain *ε*, we corrected an observed intensity *I*_0_ using the relation *I* = *I*_0_ exp(*ε*/2) assuming the homogeneous axial deformation.

Differential stress of the sample was estimated from the distortion of the Debye ring based on the lattice strain equation^41,42^ using diffraction lines 021 and 130 in olivine and 400 and 440 in ringwoodite, and the elastic constants of each phase^43–45^ (Supplementary Fig. 7). Previous studies indicated that the olivine stress measured through diffraction lines 130 and 021 tend to show values up to ~20% higher than the average values (which include other diffraction lines such as 131, 112, and 101) in the similar low-temperature plasticity regime^46,47^. Meanwhile the ringwoodite stresses measured using diffraction lines 400 and 440 show low and high-end values, respectively^10,48^. Although this may slightly affect the discussion of the relative strength of these phases in Fig. 3 and the degree of shear heating in Fig. 4, the effect is considered minor, as the degree of the overestimation is close to the error in the stress calculation.

### Acoustic emission measurements

We developed a KMA-type acoustic emission (AE) measurement system based on the previous MA 6-6 type system^49^ as shown in Supplementary Fig. 1. The total of eight lead zirconate-titanate piezoelectric ceramics (FUJICERA, PZT C-6) with resonant frequencies of 2.0 MHz (1.0 mm thickness, 3.0 mm diameter) were used as transducers. They were attached to the back truncations of the eight anvils with epoxy glue and connected to an 8ch AE receiver consisting of RF transformers, switches, and power amplifiers, with coaxial cables (Supplementary Fig. 1).

During the experiments, AE signals were recorded with an 8ch oscilloscope (Tektronix, MSO58) thorough the AE receiver by triggering with threshold amplitude of 28–44 mV after being pre-amplified with a gain of 20 dB. Data were collected for 2.0 ms with a sampling rate of 125 MHz corresponding to the duration of 8.0 ns between the data points. The intensity of waveforms was recorded with 12 bits where the resolution was ~0.1 mV. The AE waveforms were analyzed with the system of Lab-VIEW^49^. The arrival times of AEs were picked up by the AIC method^50^ after bandpass filtering of the waveforms from 200 kHz to 15 MHz. Magnitudes of AEs (M_AE_) were estimated from M_AE_ = log(*E*/*E*_0_), where *E* is the energy of AEs that is defined as a product of the sum of squares of unit amplitudes and the sampling duration. We set *E*_0_ to the minimum energy during cold compression (*E*_0_ = 1.56 × 10^−8^ V^2^ s).

The hypocenters of AEs were determined by the least-square method with arrival times. P wave velocity of 10 km/s was used for this calculation. Although the location can be estimated even if some transducers are lost during the experiments, the accuracy becomes worse along with the lost transducers. We examined it by the cold compression test with single crystalline olivine using the same pressure medium, in which a total of 25 AE events were recorded by all of 8 transducers as shown in Supplementary Fig. 8. Most of the hypocenters were located within the sample when using the arrival time data from all or seven transducers. When we analyzed the same AE events using the data only from five or six transducers, the hypocenters were shifted to the direction where the data were not used. Actually, several transducers were occasionally lost in the deformation experiments. Examples of the AE signals and locations are shown in Supplementary Fig. 9. Considering the similar trend regarding the shift of the hypocenters, most of AE events were thought to be generated within the sample.

### Microstructural observations

All recovered samples were cut into half with a low-speed saw and polished. After osmium coating with vacuum deposition, we observed their microstructure with a field emission scanning electron microscope (FE-SEM) installed at Kyushu University (JEOL JSM-7001F) and EBSD system with acceleration voltages of 15–20 kV. Additionally, parts of the samples were excavated using a focused ion beam (FIB) system, JEOL 9320FIB (Tohoku University) and HITACHI SMI3200 equipped with a microprobe system (Kochi Institute for Core Sample Research, Japan Agency for Marine-Earth Science and Technology: JAMSTEC) for transmission electron microscopy (TEM) observation. The gallium ion beam was accelerated to 30 kV during the sputtering of the sample by the FIB. The resulting foils are about 100 nm thick. A JEOL JEM-2100F field emission transmission electron microscope at Tohoku University, operating at 200 kV and equipped with a JEOL energy dispersive X-ray spectroscopy (EDS) detector system, was used for conventional TEM observation and selected area electron diffraction (SAED) pattern analysis.

### The friction coefficient of NSBs

We measured the stress of both olivine (*σ*_ol_) and ringwoodite (*σ*_rw_) in Run oldt23 where shear localization occurred by NSBs. The friction coefficient *μ* of NSBs can be directly estimated from these stress values. On the slip plane developed at the angle of *θ* from the *σ*_1_ direction under the uniaxial deformation, the normal stress *σ*_*n*_ and shear stress *τ* are described as follows:

1 $${\sigma }_{n}=\frac{{\sigma }_{1}-{\sigma }_{3}}{2}\cos 2\theta+\frac{{\sigma }_{1}+{\sigma }_{3}}{2}=\frac{\sigma }{2}\left(1+\cos 2\theta \right)+{P}_{C}$$

2 $$\tau=\frac{{\sigma }_{1}-{\sigma }_{3}}{2}\sin 2\theta=\frac{\sigma }{2}\sin 2\theta$$

where *P*_C_ is confining pressure (≈*σ*_3_) and *σ* is the differential stress (i.e., *σ*_1_-*σ*_3_). Based on the observation that the slip plane (i.e., NSBs) filled with ringwoodite was formed at *θ* ~ 45° in olivine, Eqs.1 and 2 can be modified as follows:

3 $${\sigma }_{n}=\frac{{\sigma }_{{ol}}}{2}+{P}_{C}$$

4 $$\tau=\frac{{\sigma }_{{rw}}}{2}$$

Thus, we estimated the friction coefficient (*μ* = *τ*/*σ*_*n*_) of NSBs from

5 $$\mu=\frac{{\sigma }_{{rw}}}{{\sigma }_{{ol}} \,+\, 2{P}_{C}}$$

In the runs showing unstable NSBs at lower temperatures, where *σ*_*rw*_ could not be directly measured due to the small fraction of ringwoodite, we estimated *σ*_*rw*_ from the Si diffusivity^17^ considering the local temperature increase by the latent heat release and the initial grain size in NSBs as described in the following sections.

### Local temperature increase by the latent heat of transformation

We consider that the latent heat of the transformation has an important role to weaken the NPL and cause shear localization especially at low temperatures. Here, we estimate the local temperature increase in NPL due to the latent heat release. The molar enthalpy change *ΔH*(*P, T*) of the transformation in the adiabatic condition is described as^51,52^

6 $$\Delta H\left(P,T\right)=\Delta H\left({P}_{0},{T}_{0}\right)+\int _{{T}_{0}}^{T}\Delta {C}_{P}^{{ol}-{rw}}{dT}+\int _{{P}_{0}}^{P}\Delta {V}^{{ol}-{rw}}{dP}$$

where *ΔH* (*P, T*), $\Delta {C}_{P}^{{ol}-{rw}}$, and $\Delta {V}^{{ol}-{rw}}$ is the change of enthalpy^51^, isobaric heat capacity^53^, and molar volume associated with the transformation from olivine to ringwoodite, respectively. We used the equation of state of olivine^54^ and ringwoodite^55^ for the term of integration of *ΔV*. Then, the local temperature increase *ΔT*_*L*_ is obtained from the following equation:

7 $$\Delta H\left(P,T\right)=\int _{T}^{T+\Delta {T}_{L}}{C}_{P}^{{rw}}{dT}$$

We estimated it to be ~240–270 °C with respect to the background temperature under our experimental conditions. The error of *ΔT*_*L*_ is up to ~30 °C within the seismic temperature both in the laboratory and MOW, considering uncertainties in heat capacity and molar volume^53–55^.

Note that the local temperature increase in thin lamellae quickly diminishes due to thermal diffusion. If we consider the thermal diffusivity (*κ *= 10^−6^ m^2^ s^−1^)^56^ and the length scale of λ = 10^−6^ m, the time scale for the heat dissipation is estimated to be ~10^−6^ s from the relation of $\lambda=\sqrt{\kappa t}$. It suggests that instability initiates as soon as nucleation occurs during which the heat effectively works to weaken the strength of NSBs.

### The initial grain size of ringwoodite in NSBs

In order to estimate the friction coefficient of unstable NSBs, it is important to consider the initial grain size of ringwoodite because it is expected that the shear instability occurs instantaneously when the new phase is nucleated (before the dissipation of the latent heat).

We first calculated the Avrami length *δ*_Av_ in 2D as a candidate for the initial grain size from the following equation and thermodynamic parameters^57^:

8 $${\delta }_{{{{\rm{Av}}}}}={\left(\frac{\dot{G}}{\dot{N}}\right)}^{\frac{1}{3}}$$

where $\dot{G}$ and $\dot{N}$ are the growth and the nucleation rates, respectively.

In contrast, the minimum grain size *d*_*0*_ is constrained by the energy balance for nucleation^58,59^:

9 $${d}_{0}=\frac{4\gamma }{\Delta {G}_{V}}$$

where γ is the interfacial energy (0.6 J m^−2^)^57^ and *ΔG*_*V*_ is the volumetric free energy for nucleation. We estimated *ΔG*_*V*_ from (*ΔV/V*)$\bullet$*ΔP*, where the *ΔV/V* is the volumetric strain of the transformation and *ΔP* is the overpressure from the equilibrium boundary between olivine and wadsleyite^60^. Variations of *δ*_Av_ and *d*_0_ with background temperatures at 20 GPa indicate that the *δ*_Av_ values are always lower than the minimum value *d*_0_ (=4.5–7.6 nm). Therefore, we used the *d*_0_ as the initial grain size in the discussion. The *d*_0_ value is slightly smaller than the observed minimum grain size (~10 nm) in the recovered sample (Fig. 2d).

### Frictional heating and shear strain rate on the fault plane

The temperature increase caused by the frictional slip on the fault plane (Δ*T*_*f*_) can be estimated from the slip displacement (*D*) using the following equation^34^:

10 $$\Delta {T}_{f}=\frac{D\tau }{h\rho C}\propto \frac{D}{h}\cdot \tau$$

where *h* is the thickness of the fault plane, *τ* is the shear stress on the fault (Eq. 4), *ρ* is the density of olivine, and *C* is the specific heat capacity of olivine. Based on this equation, we calculated Δ*T*_*f*_ as a function of shear stress with varying *D/h* values in Fig. 4a using *ρC* of 4 MPa/K^56^ and *G* of 100 GPa^20^. The resultant shear strain rates on the fault were then estimated from the diffusion creep of ringwoodite taking the adiabatic temperature increase of the frictional heating and the latent heat release into accounts.

## Supplementary information

Supplementary Information

Transparent Peer Review file

## Data Availability

The X-ray diffraction data obtained in this study are available in Zenodo repository under 10.5281/zenodo.13293491. Source data are provided with this paper.

## Code availability

X-ray diffraction data were analyzed using the software of IPAnalyzer and PDIndexer provided by Dr. Yusuke Seto (https://github.com/seto77)^61^.
