# Supplementary material for: The olivine-ringwoodite transformation triggers deep slab seismicity and rheological weakening
Source: Nat Commun. 2026 Apr 16;17:5264. doi: 10.1038/s41467-026-71661-z (PMC13266067; doi:10.1038/s41467-026-71661-z)
Supplement: Supplementary file 1 — Supplementary Information [file 41467_2026_71661_MOESM1_ESM.pdf]

Supplementary Information for "The olivine-ringwoodite transformation triggers deep slab seismicity and rheological weakening"

Rikuto Honda[1] *, Tomoaki Kubo[2], Masaaki Miyahara[3], Takuya Iwasato[4], Yuichiro Mori[5], Yuji Higo[6], Yumiko Tsubokawa[2], Yuta Goto[1], Akio Suzuki[7], Yuki Shibazaki[8]

[1] Department of Earth and Planetary Sciences, Graduate School of Sciences, Kyushu University, Fukuoka 819–0395, Japan

[2] Department of Earth and Planetary Sciences, Faculty of Sciences, Kyushu University, Fukuoka 819–0395, Japan

[3] Graduate School of Advanced Science and Engineering, Hiroshima University, Higashi-Hiroshima 739–8526, Japan

[4] Shin-Nippon Nondestructive Inspection Co., Ltd., Kitakyushu 803–8517, Japan

[5] Department of Earth and Planetary Science, Graduate School of Science, The University of Tokyo, Hongo, Tokyo 113–0033, Japan

[6] Japan Synchrotron Radiation Research Institute, Hyogo 679–5198, Japan

[7] Department of Earth Science, Graduate School of Science, Tohoku University, Sendai 980–8578, Japan

[8] Institute of Materials Structure Science, High Energy Accelerator Research Organization (KEK), Tsukuba 305–0801, Japan

*Corresponding author: Rikuto Honda (honda.rikuto.860@s.kyushu-u.ac.jp)

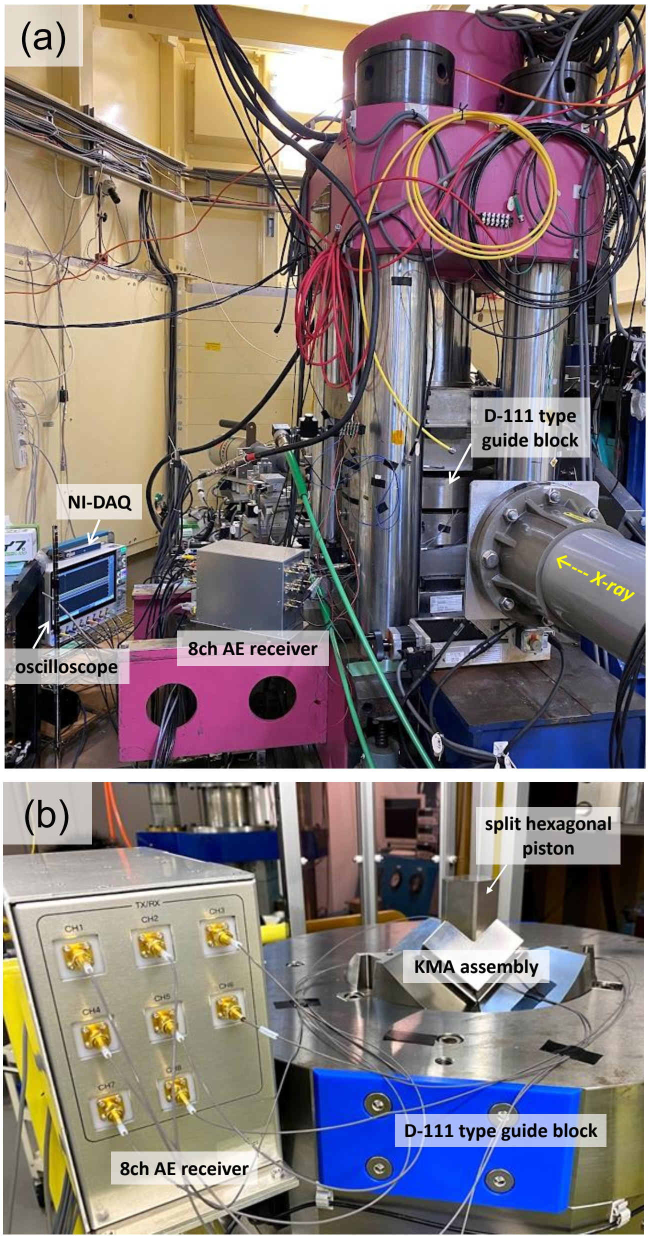

Supplementary Fig. 1. D-111 type high-pressure deformation apparatuses equipped with acoustic emission measurement system. D-111 type guide blocks are attached to 700-ton (a, MAX-III) and 1000-ton (b, QDES-II) uniaxial presses installed at the synchrotron radiation facility of the Photon Factory (PF-AR NE7A) and Kyushu University, respectively. A same 8ch acoustic emission (AE) measurement system for Kawai-type multi-anvil apparatus (KMA-type) was used for both deformation apparatuses as shown in this figure.

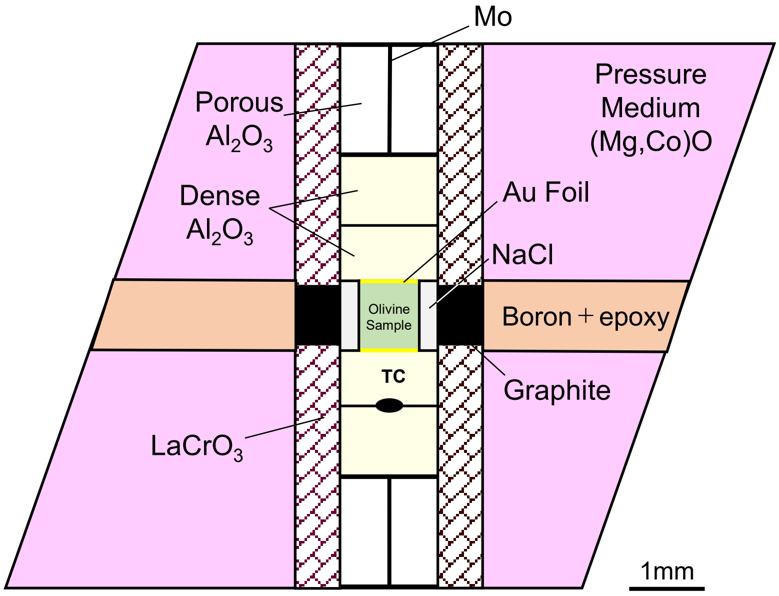

Supplementary Fig. 2. Cross section of the sample assembly.

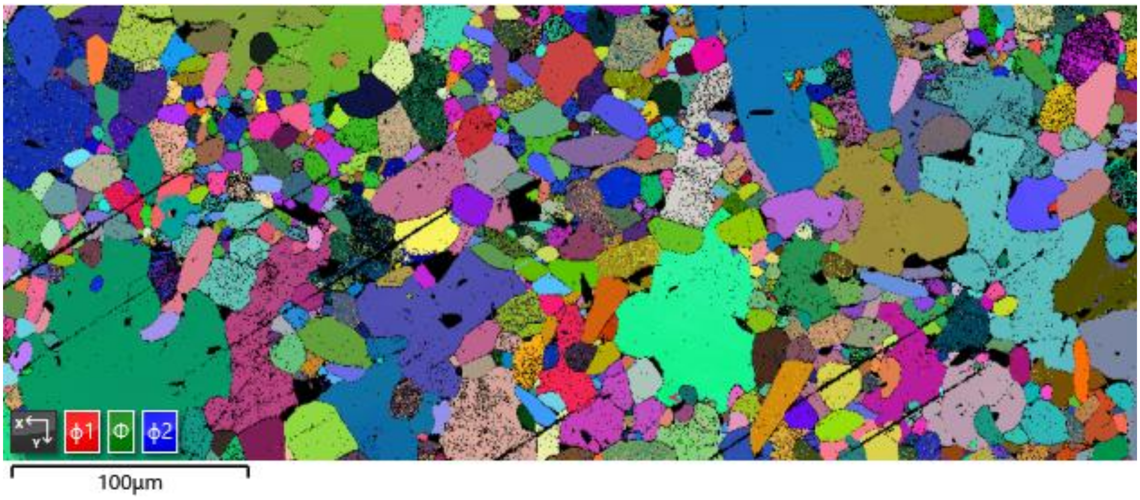

Supplementary Fig. 3. Electron back-scattered diffraction map of grain orientation in the starting material of olivine.

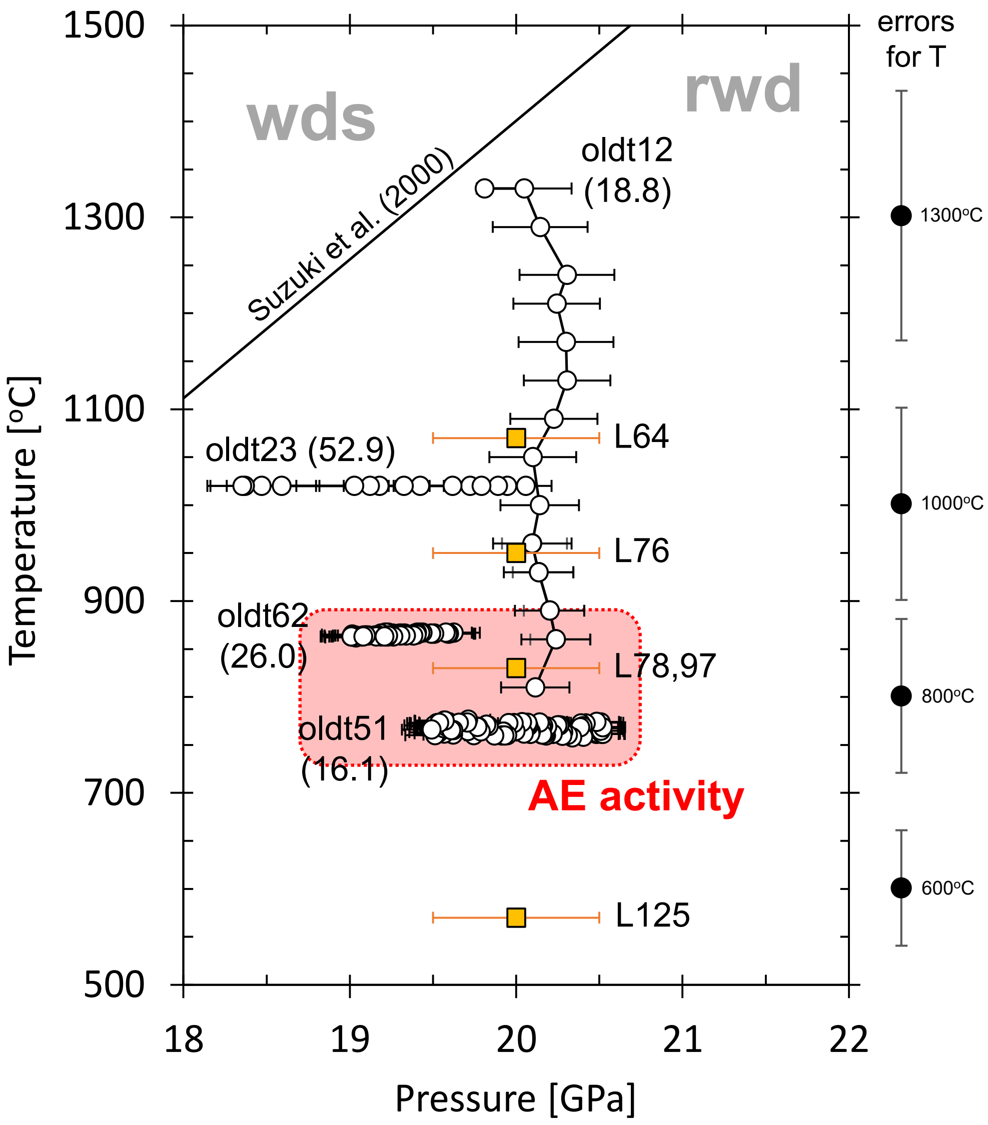

Supplementary Fig. 4. Pressure and temperature conditions for deformation experiments. In-situ X-ray observations and quenching experiments are shown in Run oldt series (open circles) and Run L series (yellow squares), respectively. Numbers in parentheses indicate the final strain in %. Acoustic emissions (AEs) were detected in the limited temperature range of 760–860°C as shown in the red hatched area. The equilibrium boundary between wadsleyite (wds) and ringwoodite[1] (rwd) is also shown. Uncertainties of pressure and temperature are shown as error bars (see Method for details).

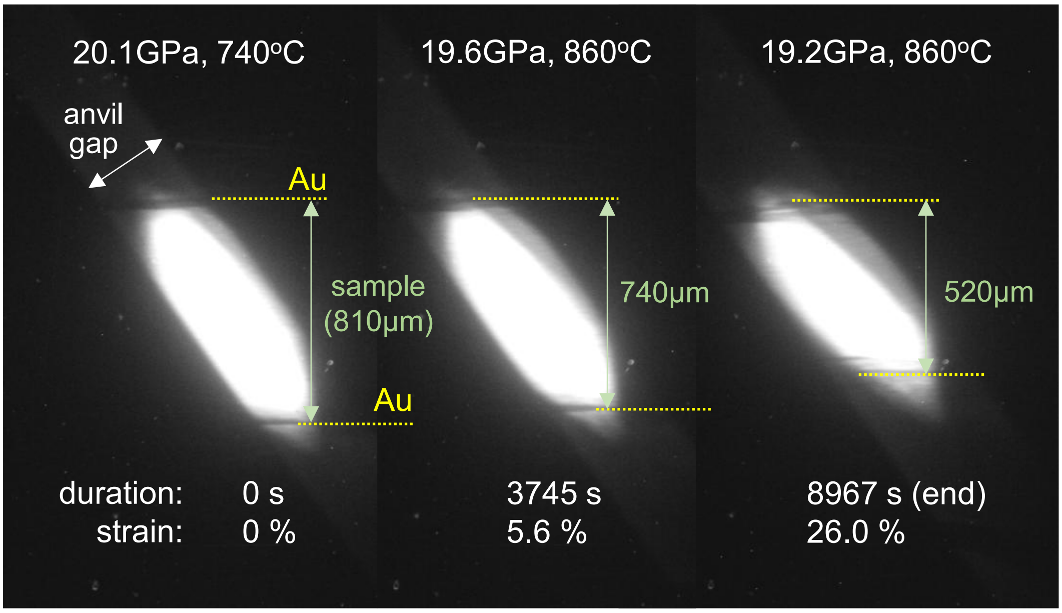

Supplementary Fig. 5. Strain measurement by X-ray radiography (Run oldt62). Note that the gold strain marker split at large strains due to the piston deformation at the boundary between the sample and the NaCl capsule. The outer markers (yellow dotted lines) indicate the sample length (green arrows).

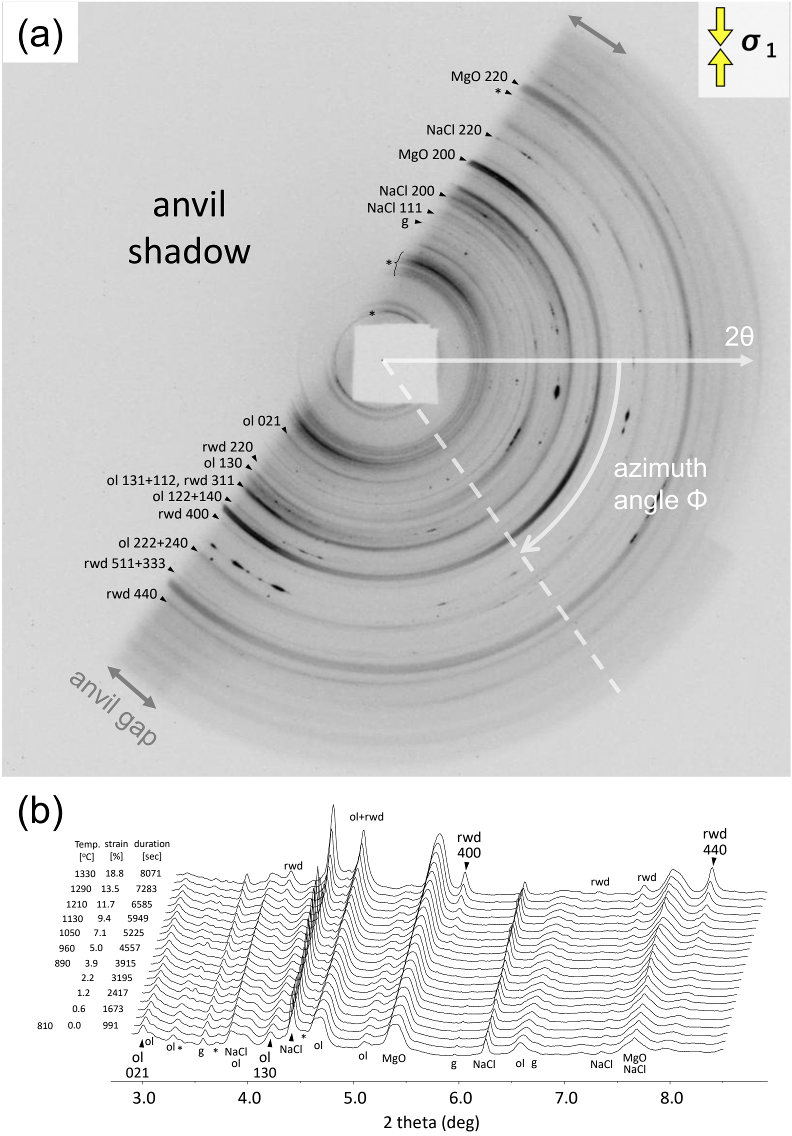

Supplementary Fig. 6. X-ray diffraction (XRD) patterns obtained during deformation and transformation (Run oldt12). (a) 2D-XRD pattern obtained at 1,210°C, 20 GPa, and the sample strain of 11.7%. The diffraction patterns in the left-upper side were hidden by a second stage anvil and in an anvil gap (shown in gray arrow) have slightly higher intensity due to no absorptions from sintered diamond anvils. (b) Changes of 1D-XRD pattern during temperature ramping. The olivine (ol) to ringwoodite (rwd) transformation was initiated at ~1,050°C and finally proceeded by ~90% at 1,330°C (g: graphite, *: pyrophyllite).

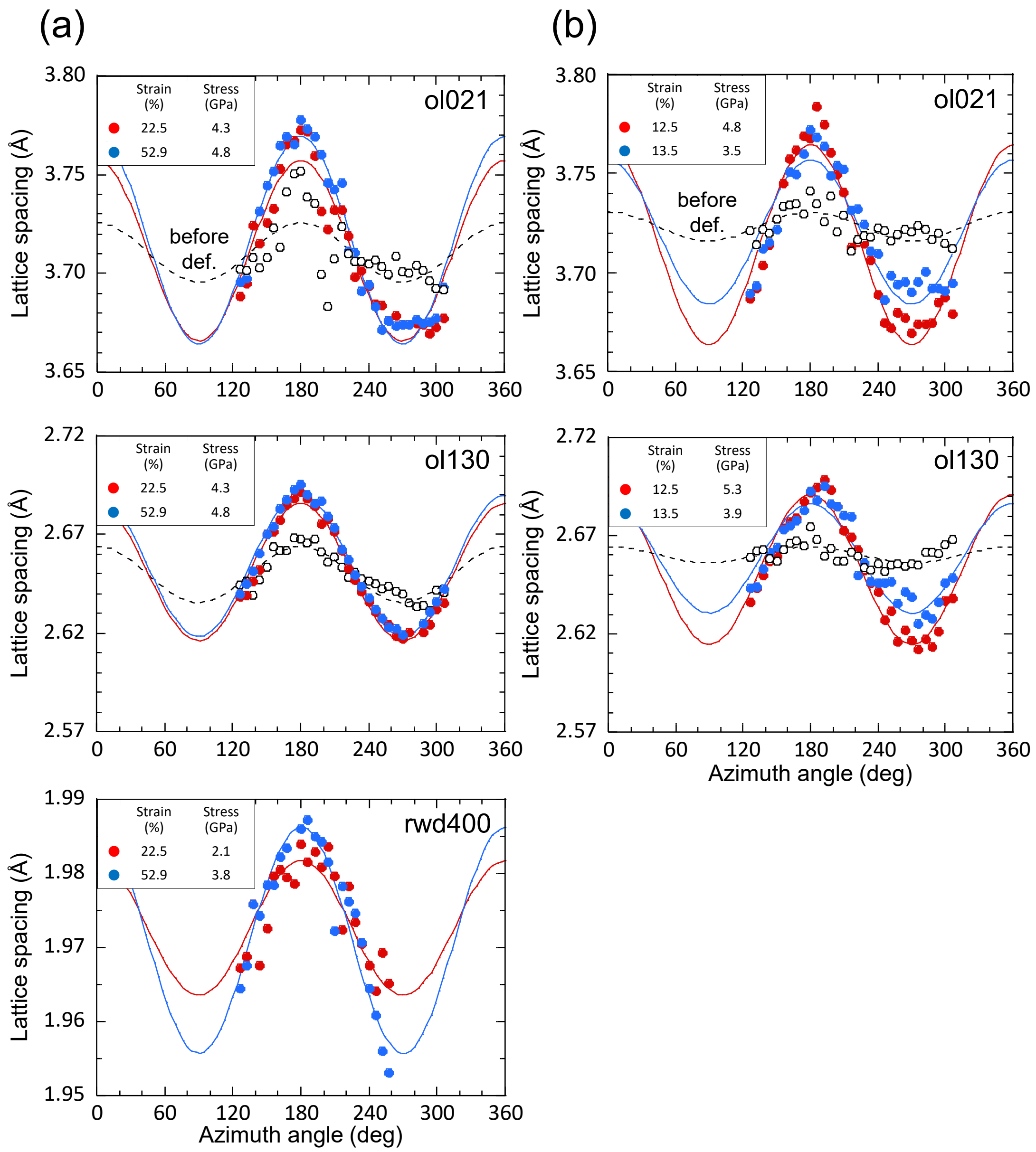

Supplementary Fig. 7. Plots of azimuthal changes of d-spacing in olivine and ringwoodite. (a) Distortions of the Debye ring at the different strains of 22.5 and 52.9% indicate steady state deformation in olivine (ol) and hardening in ringwoodite (rwd) at 1,020°C (Run oldt23). (b) Changes of the Debye ring distortions in olivine when the stress drop occurred at 860°C (Run oldt62).

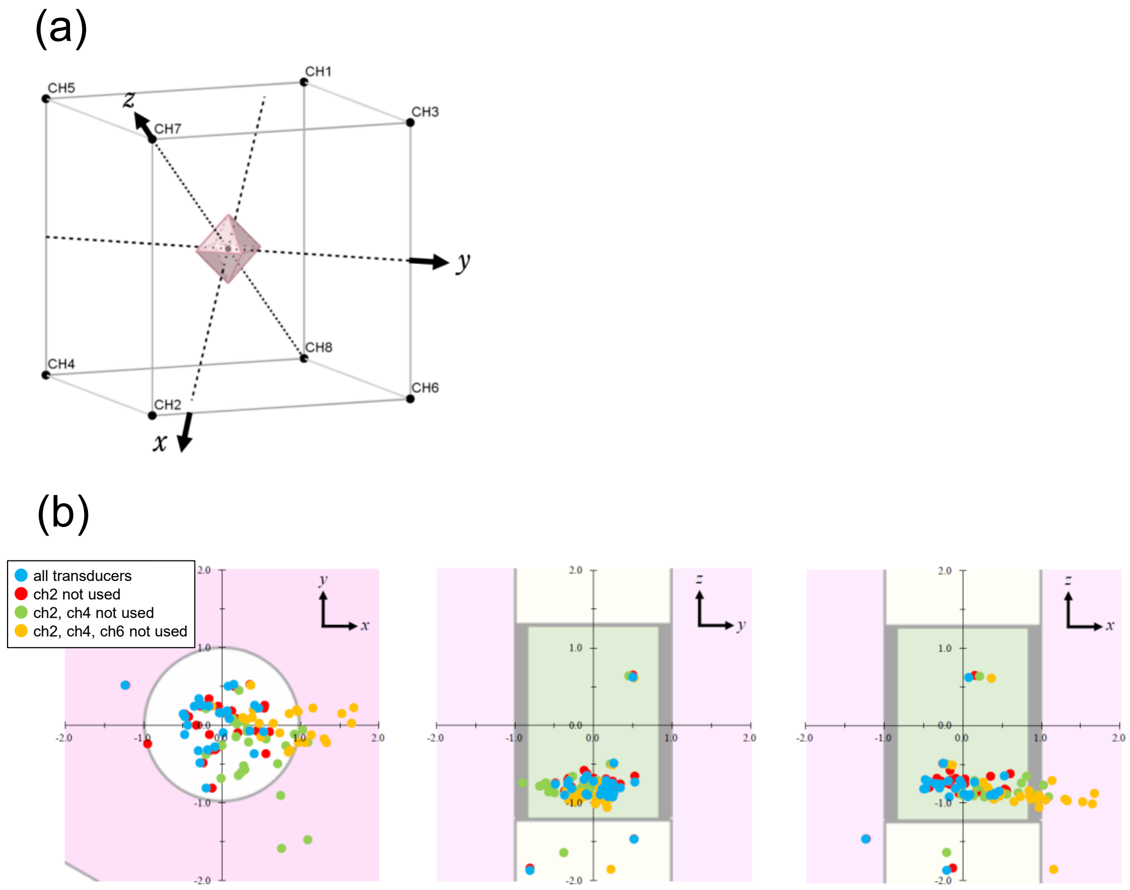

Supplementary Fig. 8. Hypocenter distribution of acoustic emissions recorded during the cold compression test. The cylindrical olivine single crystal with a height of ~2.5 mm and a diameter of ~2.0 mm was sandwiched between two dense Al_2O_3 pistons in the same pressure medium used in the deformation experiment. (a) The coordinate system in Kawai-type multi-anvil apparatus (KMA-type assembly) showing the position of each transducer. y- and z-axis correspond to the X-ray path and the σ_1 direction, respectively. (b) Plots of the locations of acoustic emission (AE) events determined by using all transducers (blue). We also analyzed the same AE events using a limited number of transducers. The hypocenter distribution was almost same when using seven transducers (red), however it was shifted to the direction where the data were not used in the case of six (green) or five (yellow) transducers.

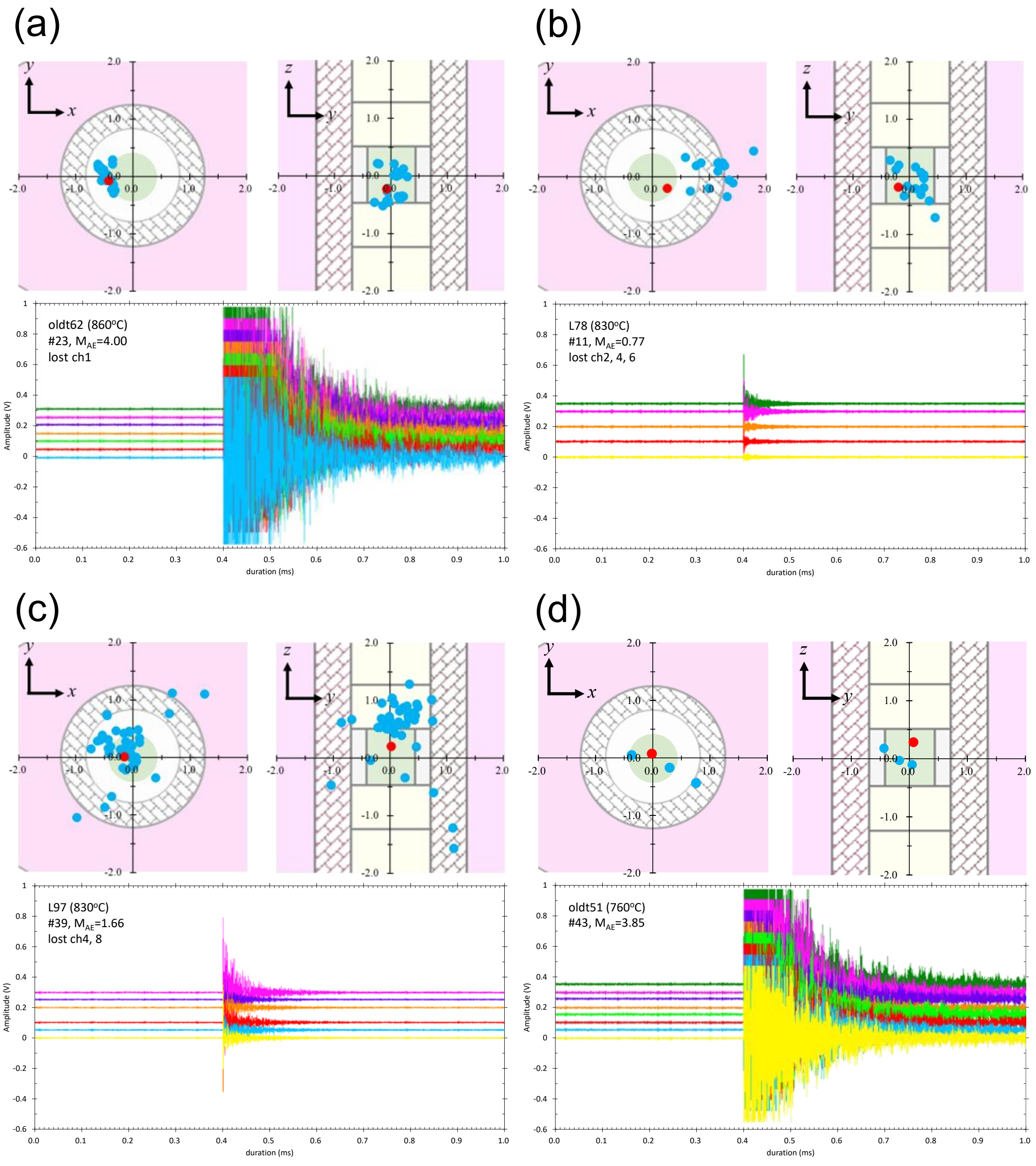

Supplementary Fig. 9. Plots of hypocenters of acoustic emissions recorded during deformation and transformation. (a) Run oldt62, (b) Run L78, (c) Run L97, and (d) Run oldt51. The location of the largest acoustic emission in each Run is shown in red, and their waveforms and magnitude (M_{AE}) are also shown.

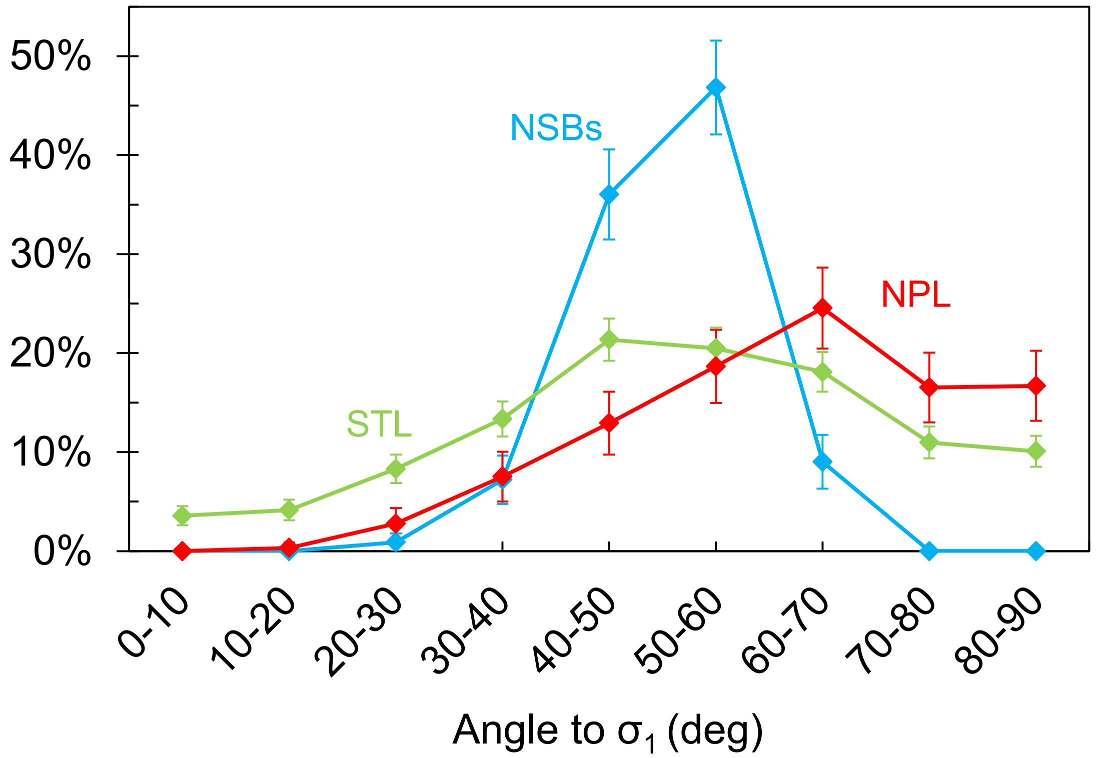

Supplementary Fig. 10. Distribution of angle of lamellae from the deformational axis. *n*=111, 611, 372 for NSBs, NPL, and STL, respectively (NSBs; nanoshear bands, STL; single-crystalline topotactic lamellae, NPL; nano-polycrystalline lamellae). Error bars indicate the binomial standard errors calculated for the frequency in each bin.

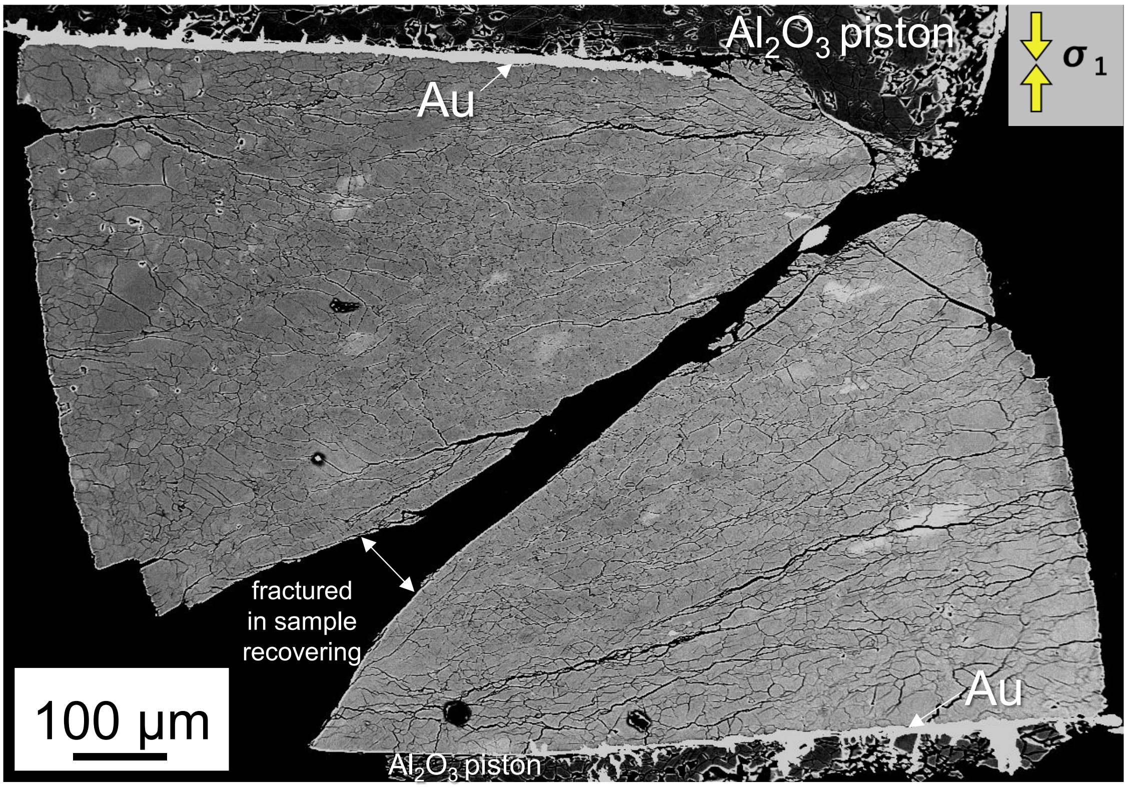

Supplementary Fig. 11. Backscattered electron image of the entire sample deformed at 860°C (Run oldt62). An open fracture inclined at ~45° to the σ_1 direction (yellow arrows) is present going through the sample. Although such fractures were observed only in the recovered sample exhibiting acoustic-emission activities, it is difficult to properly interpret microstructures of this region because all the samples were fractured during the decompression at room temperature.

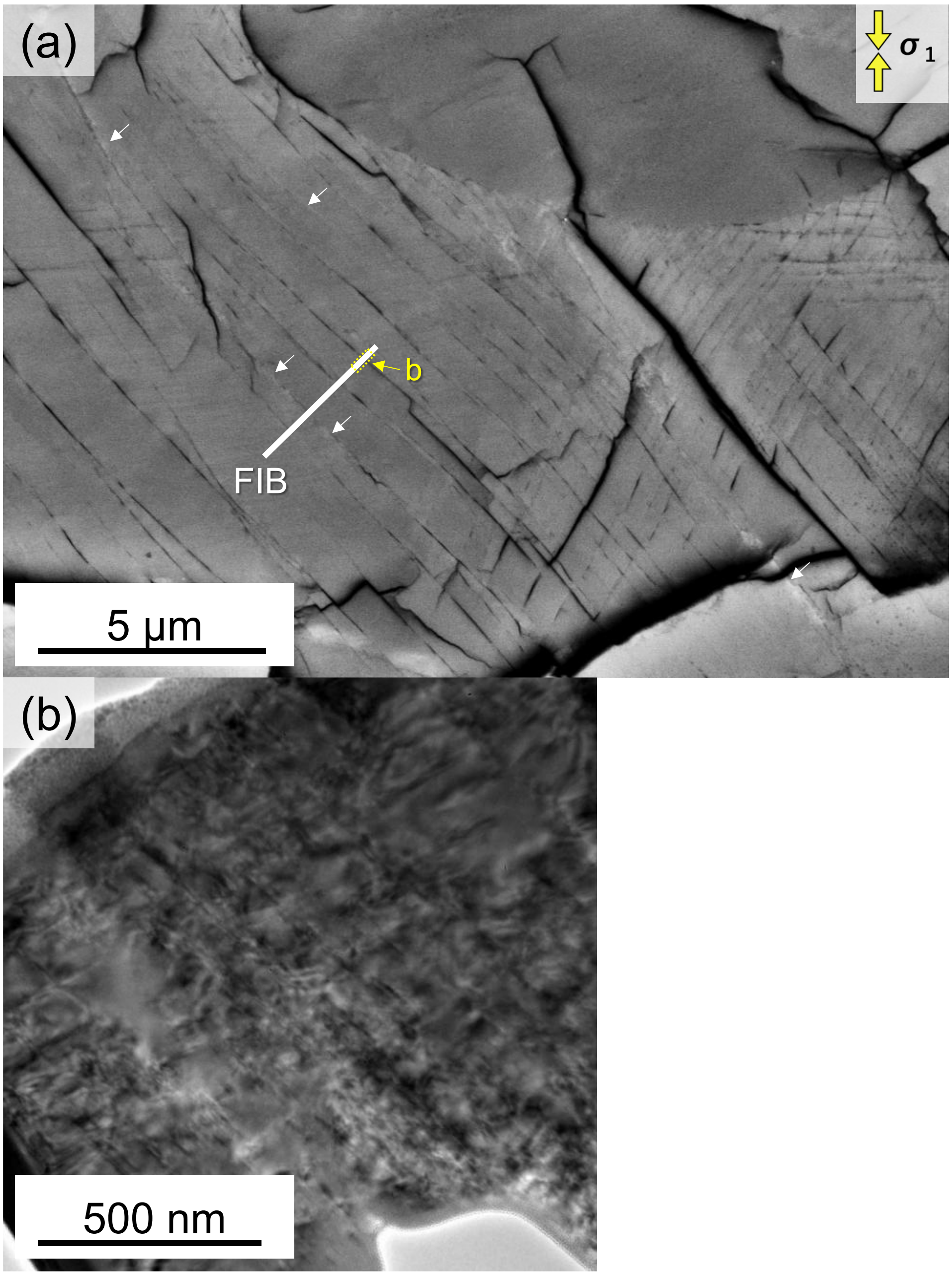

Supplementary Fig.12. Microstructures of the sample deformed in a seismogenic temperature (Run oldt51). (a) Backscattered electron image shows the deformation bands developed in the parental olivine grain, on which a small amount of ringwoodite was present (white arrows). (b) Transmission electron microscopy image of the deformation band from FIB-b in (a) showing high-density dislocations intensively tangled in olivine.

Supplementary Table 1. Experimental conditions and results.

Run No.[a]	Pressure	pre-deformation stage [b]		deformation stage			Transformed fraction	Maximum Stress		Number of AE events at High T
		Temp.	Strain	Temp.	Final strain	Strain rate		ol	rw	
	(GPa)	(°C)	(%)	(°C)	(%)	($\times 10^{-5}$ s^{-1})	(%)	(GPa)	(GPa)	
oldt12	19.8–20.6	810	2.2	810–1330	18.8	4.9(5)	88	5.4(2)	0.60(3)	---
L64	20	800	n.d.	1070	n.d.	n.d.	75 [c]	n.d.	n.d.	0
oldt23	18.4–20.3	760	7.5	1020	52.9	8.5(2)	40	5.0(2)	3.8(3)	---
L76	20	760	n.d.	950	n.d.	n.d.	70 [c]	n.d.	n.d.	0
oldt62	19.0–20.1	740	4.4	860	26.0	4.1(2)	little [c]	5.7(4)	n.d.	24
L78	20	760	n.d.	830	53 [c]	5.9 [c]	little [c]	n.d.	n.d.	15
L97	20	760	n.d.	830	n.d.	n.d.	little [c]	n.d.	n.d.	44
oldt51	19.2–20.5	---	---	760	16.1	2.8(1)	little [c]	6.2(3)	n.d.	4
L125	20	---	---	570	n.d.	n.d.	no	n.d.	n.d.	0

All deformation experiments were conducted with a constant anvil displacement rate of ~300 μm/h for 2.5h (~750 μm in total) except for Run oldt12 (660 μm in total). n.d. indicates that these values were not determined. The uncertainties in temperature are ±10% for all experiments, whereas those in pressure are ±0.1–0.3 GPa for in-situ experiments and ±1 GPa for quenching experiments. Uncertainties for other quantities are indicated in parentheses.

[a] "oldt" series are in-situ X-ray observation, and "L" series are quenching experiments.

[b] Most samples were pre-deformed for the first 1h (anvil displacement of ~300 μm) at lower temperatures before the transformation.

[c] Strain and transformed fraction were estimated from the recovered sample.

Reference

1. Suzuki, A., et al., In situ determination of the phase boundary between wadsleyite and ringwoodite in Mg_2SiO_4. *Geophys. Res. Lett.* **27**(6), 803–806 (2000).
