# Supplementary material for: The olivine-ringwoodite transformation triggers deep slab seismicity and rheological weakening
Source: Nat Commun. 2026 Apr 16;17:5264. doi: 10.1038/s41467-026-71661-z (PMC13266067; doi:10.1038/s41467-026-71661-z)
Supplement: Supplementary file 2 — Transparent Peer Review file [file 41467_2026_71661_MOESM2_ESM.pdf]

nature portfolio

Peer Review File

The olivine-ringwoodite transformation triggers deep slab seismicity and rheological weakening

Corresponding Author: Mr Honda Rikuto

This file contains all reviewer reports in order by version, followed by all author rebuttals in order by version.

Version 0:

Reviewer comments:

Reviewer #1

(Remarks to the Author)
Hobda et al. present a series of deformation experiments on olivine transitioning into ringwoodite under high-pressure and temperature conditions (including acoustic emission experimental data), followed by textural analysis of the recovered samples. Their results indicate that the superplasticity of the newly formed ringwoodite plays a significant role in the deformation behavior of slabs in the Mantle Transition Zone (MTZ). Overall, I find the experimental results compelling, and the observations are largely robust, though a few minor points require further clarification, which I will outline below. However, the central hypothesis driving this study is not novel, as it was previously proposed in Mohiuddin et al. (2020, Nature Geoscience). The main strength of the present work lies in its more comprehensive approach, which provides solid evidence for the temperature-dependent transition and weakening of MTZ slabs. Below, I offer several minor comments and suggestions for revision:
1. Deformation Geometry (Uniaxial Deformation vs. Natural Slab Penetration): The uniaxial deformation geometry used in this study differs from the natural geological process in which slabs penetrate into the mantle. How might this difference affect the applicability of the experimental results to real-world slab dynamics?
2. Composite Slabs: Slabs are not composed solely of olivine-dominated peridotite. Liu and Zhang (2015, EPSL) discussed strain/stress buildup at the crust-mantle boundary in composite slabs. How does this model apply in the case of composite slabs with olivine-rich lithospheres and olivine-poor crusts? A more detailed discussion of how this model translates to the heterogeneous composition of real slabs would be beneficial.
3. Irrelevance of Meteorite Data (Lines 97-98): While it is interesting that L6 shocked meteorites also exhibit two types of ringwoodite lamellae, this observation does not appear directly relevant to the primary research questions addressed in the paper. I recommend removing this sentence and its associated references to keep the focus on the key issues.
4. NPL and STL Angles (Line 100): The statement regarding the orientation of NPL at high angles to the deformation axis requires statistical backing. A figure presenting the statistical distribution of NPL and STL angles would strengthen this claim and improve the manuscript's clarity.
5. Latent Heat Comparison (Lines 193-201): The authors discuss latent heat and compare germanate to magnesium silicate systems, but the assertion that latent heat release in germanate systems is more significant lacks appropriate citations (Line 196). Additionally, the connection between the discovery of nanocrystalline fine grains in germanate systems, the orientation of faults at 45 degrees relative to the sigma1 direction, and greater latent heat release in germanates is unclear. This section could benefit from clearer articulation and reference support to bolster the argument.
6. Geological Implications (Lines 239-255): The application of the experimental results to real geological examples could be expanded. Deep earthquakes occur in only a limited number of locations globally, and thus, a broader synthesis of observations would improve the implications section. I recommend including a table or graph summarizing the findings, their implications, and their applications to better illustrate how the experimental results relate to deep-earth phenomena.
7. Re-emphasizing Mohiuddin et al. (2020): While the authors reference Mohiuddin et al. (2020), the significance of this prior study could be highlighted more. I understand the authors did not intend to downplay its contribution, but after reading this manuscript, I was left with the impression that the idea of grain-size reduction-induced weakening for deep-focus earthquakes was novel to this study. I recommend a revision that better acknowledges the importance of this prior work, ensuring that the manuscript appropriately credits the foundation laid by Mohiuddin et al. (2020).
In summary, the manuscript presents interesting and robust experimental results with significant implications for the study of slab dynamics in the MTZ. However, it would benefit from revisions to clarify and strengthen the discussion, particularly regarding its novelty, the application of results to composite slabs, and the connection to prior work.

Reviewer #2

(Remarks to the Author)
The authors present an experimental work on the long-standing issue of the origin for deep earthquakes occuring within the transition zone, that may be caused by transformation of metastable olivine to its high-pressure polymorph ringwoodite. They use high pressure large-volume experiments, coupled with ex-situ characterization of the samples microstructures, and with in-situ x-ray diffraction and acoustic emission measurements which provide insights into the stresses and brittle character of the deformation. From these results they calculate frictional properties of nano-polycristalline ringwoodite shear bands and investigate their potential to become « seismic » due to latent heat release and frictional shear heating.
This topic has been addressed before using similar coupled methods - a number reference works are duly cited in the introduction.
However, this study stands out as an extensive experimental work on the system using the actual olivine composition in the mantle - as opposed to previous ones at lower pressures or using analogs. The deformation experiments by themselves are extremely challenging to carry out at these pressures and the dataset seems pretty unique. The study also includes documentation of microstructural relationships for different shear bands types, using microscopy, on samples deformed at these pressures of 20 GPa, which has been seldom made I beleive.
The extrapolation to nature is more speculative and contains some weaknesses (comment (3)). The publication should be of great interest to communities working on the earth and planetary interiors from mineralogists to modelers and seismologists.
The manuscript is well written and well organized. The figures are informative and clear.
I would recommend publication of this work, with modifications.

Here are some reservations or questions on this manuscript, (3) and (2) being the most important ones :

1) The presentation of the coexistence of weak zones and strongly stressed zones in the MTZ as a « paradox » is simplistic to me (abstrast, beginning of the introduction). Line 49 on the stagnation of slabs : how about the influence of density contrasts ?

2) Lines 127-131 (and lines 210-211): the flow law calculation using diffusivity remains unclear. It seems the flow laws for diffusion used here have been established at higher grain sizes (>micron) and higher temperatures (1400 to 1600°C) (reference 16). Can these be extrapolated down to 20 nm grain sizes and 1000°C (figure 3b) ? Or even farther, to the conditions of the lowest temperature runs (fig. 3c,d) ?
With these uncertainties, it is difficult to trust that the calculated (huge) difference in stress caused by variations of grain sizes from 20 nm to 27 nm, is significant (line 131). Moreover, the grain sizes range in the bands, is probably wider than 20 to 27 nm in the actual sample.
Finally, can this flow be called superplastic (line 126 - a reference would be welcome, and lines 136, 211) ? What is the relationship with possible superplasticity with grain boundary gliding or sliding ?

3) I believe heat dissipation cannot be overlooked and should be addressed in the main text (ie. the lines 533-537 should be with Figure 4 and lines 221-237). To me, a proper discussion between timescales for heat dissipation, that occurs on the microsecond, and the timescales involved for the processes that produce shear strain, is needed. This affects the overall significance of figure 4 and the associated discussion.

In the same line the extrapolation of the experimental results to earthquakes is not so strong here, based on a simple ratio D/h ; that does take into account possible changes in the instabilities propagation mechanisms, from the sample scale, to the fault/earthquake scale.

4) Stress in olivine are measured through diffraction lines 130 and 021, which usually are on the high-end of the stresses calculated from the different crystallographic planes in olivine. The stress in the aggregate may be lower than that (e.g. Nishihara et al, 2008, works by Burnley and collaborators showing the need for EVPSC models, ...). What would be the consequences for the calculations ?

5) The number of AE events recorded (Extended table 1) is very low, e.g. relative to studies like Schubnel et al, 2013. On the other hand, Schubnel et al. did not observe stress drops from the diffraction patterns. Could these differences be due to different mechanisms, or different experimental strategies ?

6) lines 213-220 : can this latent heat release hypothesis really be applied to a couple of lenses of ringwoodite, such as those for run Oldt51 (last extended figure), that do not form a continuous band ?

7) On the stress measurement geometry : It would be useful to add arrows showing where the maximum stress is on the 2D diffraction pattern figure 6a.
The authors made a correction for absoption of the sample width change, with increasing strain. Making a correction for the sintered diamond anvil absorption may be more important, since it seems to affect the diffraction intensities with respect to the azimut angle. Or is the olivine texture, relative to the maximum stress, unusual ?

Line by line comments:

Line 53 : I do not understand; should 'cause' be replaced by 'undergo' ?

Line 124 : « the stress of ringwoodite remained »

Line 138 : low temperature plasticity could be more appropriate than « Peierls ». The activation volume chosen is on the very high-end side.

Line 209-210: not so easy to understand, maybe try reworking the sentences.

Line 214 « at the instant when » english can probably be improved here

Line 243 : « location of foci of deep ...» : location of deep earthquakes ?

Reviewer #3

(Remarks to the Author)
The formation mechanism of deep-focus earthquakes has been a long-standing question for rock physics and seismology communities for several decades. This work used only olivine as a starting material and focused on the in situ observations for the deformation and phase transformation of olivine to ringwoodite at pressure and temperature conditions corresponding to the bottom of MTZ. The pressure-induced formation of nanoshear bands (NSBs) has been reported from previous reports (Burnley et al. 1991; Schubel et al. 2013; Wang er al. 2017) using Mg2GeO4 and hazburgite-like composition. The experimental method used synchrotron X-ray together with acoustic emission for deformation and rupture detection have also been demonstrated previously for deep-focus earthquake studies. This work is similar as report from Burnley et al. (1991), Schubel et al. 2013 and Wang er al. 2017 but provides with slightly different analyses (Fig. 2) in SEM and TEM from their quenched products. What makes the contribution different from others? The authors used mantle dominate olivine at P-T conditions close to subducted cold slabs. They clearly demonstrate the formation of NSBs associated with the uniaxial compression direction and explain the formation of conjugated faulting zone. Importantly, they suggest the appearance (nucleation) of ringwoodite plays a key role for the formation of weak plane and thus causes the shear instability. The shear strain rate with respect to shear stress shows reasonable explanations for the superplastic flow in ringwoodite that may be related to subducted slabs.

Besides, there are some minor questions needed to be answered.
Line 94-97 describes the interesting orientation of rwd grown under high P-T in the Fig. 1 with fine grains in central layer and outside with elongated and roughly perpendicular oriented grains. Any more detailed photos to show this?
Line 129 "…hardening behavior at constant temperature can be explained by the changes in grain size from 50 nm to 200 nm, and from 131 20 nm to 27 nm, respectively…" seems confusion. The constant temperatures shown in Fig 2a and 2b did not clearly exhibit stress hardening behavior, especially for rwd440. Furthermore, what's the explanation for the drop of strength on rwd400 at strain rate of 34-45%?

Line 138 state the high dislocation density in olive. Can the authors elaborate to explain this explicitly? It's a bit difficult to understand in Fig. 2b.

Why rwd440 shows in Fig 3a but rwd400 shows in Fig 3b?

Line 210 shows the definition of superplastic flow (i.e. diffusion creep) that needs to be defined in early paragraph (Line 126).

Line 222, please explain the velocity-strengthening behavior.

Version 1:

Reviewer comments:

Reviewer #3

(Remarks to the Author)
This work emphasizes their findings on the formation of stress-induced NPL that may cause grain-size sensitive creep and thus trig the deep-focus earthquake at low temperatures. The authors have answered most of the questions raised by the reviewers and provided an updated revision for better presentation of their work. I recommend publishing this work. However, there are still two minor issues to be resolved.

Reviewer 1 asked the authors to provide a figure presenting the statistical distribution of NPL and STL angles to clarify the claim which I also agree. However, the newly added Fig. 10 did not show the errors in this plot that may weaken the argument.

In Fig.4a, the shear heating temperature plot is helpful for showing the thermal instability but it is difficult to read from the plot. For example, Line 263-264 states D/h ~1.5 …is expected to increase the temperature by ~750–940°However, it is hard to read this temperature from Fig. 4a. Furthermore, the TZ temperatures are expected in the range of 600-2000 0C. It is thus suggested to show the temperature scale not exceeding 3000 (or 2500) 0C to allow readers to read the meaningful shear

heating temperatures.

Reviewer #4

(Remarks to the Author)
Review of manuscript “The olivine-ringwoodite transformation triggers deep slab seismicity and rheological weakening” by Honda et al., submitted to Nature Communications

This study reports novel experimental measurements that characterize the coupling between the olivine-to-ringwoodite phase transformation and deformation processes, highlighting their significance for understanding deep-focus seismicity and mechanical weakening of subducting slabs under mantle transition zone conditions. To accomplish this goal, the authors implemented a comprehensive experimental approach that integrates high-pressure large-volume deformation experiments with in situ X-ray diffraction and imaging to quantify stress–strain behaviour, acoustic emission monitoring, and detailed ex situ microscopic examination of the resulting microstructures. The key finding is that stress-induced formation of nanopolycrystalline ringwoodite initiates deep-focus seismicity under low-temperature conditions, while the subsequent bulk phase transformation results in homogeneous rheological weakening at higher temperatures.

This conclusion is not entirely new, as it has been proposed in several previous studies employing essentially similar methods on analogue materials. However, the significance of the present work lies in the fact that the measurements were performed directly on Mg_2SiO_4 olivine under mantle transition zone P-T conditions—an important experimental achievement. The study therefore provides compelling confirmation that the mechanisms identified in analogue systems are also operative in natural compositions. This in itself justifies the publication of this work, which will be of interest to a wide audience within the deep earth scientific community.

I did not evaluate the initial version of the manuscript, but I carefully reviewed the modified version and the response to the comments of the previous reviewers. I found the conclusions to be robust and well supported by the in situ measurements and the post-mortem microscopic analyses of the samples. The methods are well developed, and the article is clearly and effectively illustrated (with useful extended data figures), in part thanks to the reviewers’ constructive feedback.

Of course, extrapolation to natural conditions is complex given the mineral heterogeneity of the plates, their stress state, grain sizes, the role of heat dissipation, etc…, and may be open to debate. However, these points are beyond the scope of this study, as the authors point out.

My only reservation concerns the treatment of uncertainties in the sections on the interpretation of the local temperature increase due to latent heat of transformation, the initial grain size of ringwoodite in the NSBs, as well as friction heating and the rate of shear deformation on the fault plane. Including a more detailed discussion that accounts for the uncertainties in the different parameters would provide valuable insight into the sensitivity of the conclusions to these factors.

Apart from this comment, this revised version fully meets the scientific and presentation standards required for publication in Nature Communications with only minor adjustments.

Reviewer #5

(Remarks to the Author)
Summary

The purpose of this article is to share recent findings on the nature of rheological weakening, shear instability, and the effects of transformation of metastable olivine to ringwoodite within a subducting slab in the mantle transition zone, as well as to explore the contribution of grain-size sensitive creep to these large-scale behaviors. The details of these processes have not previously been explored or characterized in detail experimentally. The authors aim to do this using high-pressure, high-temperature in-situ deformation experiments and acoustic emissions measurements.

Samples are compressed up to 20 GPa at room temperature, and then uniaxially deformed between 570 – 1,330 °C with a constant anvil displacement rate using a D-111 type deformation apparatus.

Major findings include the formation of high-pressure phases nano-polycrystalline lamellae (NPL), single crystalline topotactic lamellae (STL), and nanoshear bands (NSBs), all of which are thinly nucleated ringwoodite phases dispersed in certain areas of the sample or throughout the sample, respectively. These phases are key in rheological weakening via grain-size sensitive creep. Acoustic emission (AE) measurements and XRD were used to further characterize the coupling of phase transformation and deformation by seismicity. This work supports previous work suggesting that the aforementioned microstructural processes lead to slab weakening and potentially deep earthquakes.

Strengths

The work is novel in its effort to experimentally explore the coupling of deep seismicity and the high-pressure phase transformation of olivine. The experiments carried out are detailed. The results are well presented with clear diagrams.

Care is taken to distinguish the results of deformation by temperature and pressure, because some of the processes of interest are kinetically controlled.

The practice of in-situ deformation experiments with X-ray synchrotron presents trustworthy quantitative results, further bolstered by the cross-correlation of the XRD and AE results.

The development of a new KMA-type acoustic emission measurement system may be useful for future AE studies.

Figure 1A clearly portrays the NPL and STL deformation features. The figure would benefit from having NSBs on the photo as well, for comparison.

Authors present a strong qualitative analysis of NPL's contribution to grain-size sensitive creep, including Figure 3, which highly motivates future quantitative studies on the topic.

Challenges

The functional distinction between nanoshear bands (NSBs) and the lamellae (single-crystalline topotactic lamellae (STL) and nano-polycrystalline lamellae (NPL)) is a bit unclear. I suggest providing further clarification in the section titled "Formation of nanoshear bands at medium T".

The values in Extended Data Table 1 would benefit from adding uncertainties to allow readers to evaluate data spread.

The work addresses the question of deep seismicity in the metastable olivine wedge, which is relatively cold compared to the surrounding mantle and outer slab. Some experiments explored these lower temperatures, but no ringwoodite or acoustic emissions were formed in these runs. Latent heat was suggested as a source of the additional heat needed to facilitate these reactions (line 297), but without extrapolating, this result is worrisome.

There did not appear to be consideration for other kinetic mechanisms besides the major grain-size sensitive creep. The study could be supported by a brief consideration of other diffusion regimes.

Line 221: What is "stick-slip behavior"? The authors could introduce this concept to the reader.

No mention is made of the effect of water. Water has a significant effect on deformation and grain growth. Is the mantle assumed to be totally dry? Did the authors attempt to measure the water contents in the samples used?

Line 153: Was there any flow law determined from the experiments? No scaling law from laboratory to geophysical time scales is discussed. The authors should present a flow law from the experiments and discuss how the results can be projected from laboratory to geological timescales where typical strain rates are of the order of 10-15 s-1.

The phrase "On the other hand" is used repeatedly in the manuscript at the start of sentences/paragraphs.

Lines 244 – 248 and Lines 262 – 264: Two concepts are discussed by the authors, which can be a little confusing to the reader: Latent heat release due to phase transformation (reducing friction), and frictional heating. They suggest that latent heat can increase the temperature by 200 – 220 oC while frictional heating can increase in temperature by ~750 – 940 °C as a result of slip. The authors need to give clarity on the overall effect of temperature from these two concepts.

Before the manuscript can be accepted for publication, I suggest that the above concerns be addressed.

Reviewer #6

(Remarks to the Author)
I co-reviewed this manuscript with one of the reviewers who provided the listed reports. This is part of the Nature Communications initiative to facilitate training in peer review and to provide appropriate recognition for Early Career Researchers who co-review manuscripts.

Version 2:

Reviewer comments:

Reviewer #5

(Remarks to the Author)
The authors have responded satisfactorily to all my comments and concerns. I therefore recommend the manuscript for publication.

Open Access This Peer Review File is licensed under a Creative Commons Attribution 4.0 International License, which permits use, sharing, adaptation, distribution and reproduction in any medium or format, as long as you give appropriate credit to the original author(s) and the source, provide a link to the Creative Commons license, and indicate if changes were made.
In cases where reviewers are anonymous, credit should be given to 'Anonymous Referee' and the source.
The images or other third party material in this Peer Review File are included in the article's Creative Commons license, unless indicated otherwise in a credit line to the material. If material is not included in the article's Creative Commons license and your intended use is not permitted by statutory regulation or exceeds the permitted use, you will need to obtain permission directly from the copyright holder.
To view a copy of this license, visit https://creativecommons.org/licenses/by/4.0/

NCOMMS-24-66673-T

Title: The olivine-ringwoodite transformation triggers deep slab seismicity and rheological weakening

Authors: Rikuto Honda et al.

Late Update: Augst 10th, 2025

Responses to Reviewers' comments

(Authors' responses and the line numbers in annotated revised manuscript are shown in red.)

Reviewer #1 (Remarks to the Author):

Honda et al. present a series of deformation experiments on olivine transitioning into ringwoodite under high-pressure and temperature conditions (including acoustic emission experimental data), followed by textural analysis of the recovered samples. Their results indicate that the superplasticity of the newly formed ringwoodite plays a significant role in the deformation behavior of slabs in the Mantle Transition Zone (MTZ). Overall, I find the experimental results compelling, and the observations are largely robust, though a few minor points require further clarification, which I will outline below.

However, the central hypothesis driving this study is not novel, as it was previously proposed in Mohiuddin et al. (2020, Nature Geoscience). The main strength of the present work lies in its more comprehensive approach, which provides solid evidence for the temperature-dependent transition and weakening of MTZ slabs. Below, I offer several minor comments and suggestions for revision:

We sincerely appreciate your review and helpful comments, which provide valuable insights for improving our manuscript and offer alternative perspectives. We have carefully reviewed your comments and revised the manuscript according to your suggestions, as explained in the following sections. However, we would like to clarify potential misunderstandings regarding the significance of our work in comparison to the earlier work by Mohiuddin et al. (2020). We will address this issue here.

Mohiuddin et al. have demonstrated that fine-grained ringwoodite, transformed from olivine, is rheologically weak due to superplasticity, which leads to slab weakening. However, it is important to note that the main focus of our study is not slab weakening, but rather deep earthquakes, which were not addressed in Mohiuddin et al. In fact, they concluded with the statement: "The stability of deformation of such a material needs further investigation in connection to deep earthquakes". This clearly indicates that the issue of shear instability triggering deep earthquakes was not explored in their experimental study and remains an unresolved critical issue. To address this, we have developed several new techniques, such as D-111 apparatus and KMA-type AE measurement system, which enabled us to solve the paradox of why deep earthquakes occur in weak slabs.

We hope this explanation helps clarify the critical relationship between our study and the earlier work, both of which are indispensable for the advancing our understanding of the deep slab dynamics.

While this issue may have arisen from insufficient explanations in the original manuscript, we are confident that we can address it through by improvements based on the reviewers' comments.

1. Deformation Geometry (Uniaxial Deformation vs. Natural Slab Penetration): The uniaxial deformation geometry used in this study differs from the natural geological process in which slabs penetrate into the mantle. How might this difference affect the applicability of the experimental results to real-world slab dynamics?

Previous studies on seismicity (e.g., Neto et al., 2024 ESS) and slab dynamics simulation (e.g., Ishii et al., 2024 EPS) have indicated that the stress field generating deep Earthquakes in the vicinity of the metastable olivine is down-dip compression, in which shear faulting (instability) occurs in a uniaxial stress field. Therefore, we think that our uniaxial deformation experiments can be applied directly to the natural slab condition where the deep-focus earthquakes occur. Furthermore, we also agree with the reviewer's comments and consider that the other deformation geometries such as shear deformation are quite important to understand the frictional evolution along the high-pressure faults. For example, we found that shear deformation initiates along the NPL and their precursor (deformation lamellae) in the uniaxial stress field, where the strain remains rather small up to ~0.3. It is indispensable to investigate evolution and stability of these shear localizations in large shear strains in the future work.

2. Composite Slabs: Slabs are not composed solely of olivine-dominated peridotite. Liu and Zhang (2015, EPSL) discussed strain/stress buildup at the crust-mantle boundary in composite slabs. How does this model apply in the case of composite slabs with olivine-rich lithospheres and olivine-poor crusts? A more detailed discussion of how this model translates to the heterogeneous composition of real slabs would be beneficial.

Thank you for introducing the reference providing an important perspective. The composite slabs generate higher stress levels in the subducting slab at MTZ due to the differential contraction. In our model, the higher stress in metastable olivine wedge (in the center of the slab) facilitates the occurrence of instability, as shown in Fig.4. Therefore, instability can be triggered more easily under higher stress conditions inferred from the composite slab than from a purely olivine-dominated slab. We inserted sentences related to these topics into lines 317–318.

3. Irrelevance of Meteorite Data (Lines 97-98): While it is interesting that L6 shocked meteorites also exhibit two types of ringwoodite lamellae, this observation does not appear directly relevant to the primary research questions addressed in the paper. I recommend removing this sentence and its associated references to keep the focus on the key issues.

Mechanisms of high-pressure transformations observed in heavily shocked meteorites have provided

important insights into deep slab phenomena (e.g., Chen et al., 2004 PNAS; Miyahara et al., 2021 PEPS; Tomioka 2007, JMPS; Tomioka and Miyahara 2017 MaPS). Although there are significant differences in timescales between these processes, we can observe how minerals behave in response to the simultaneous application of high pressure and deformation. Previous high-pressure studies that did not apply deformation reported only the STL and suggested its important role in kinetics and rheology. It has been puzzling that both STL and NPL exist in shocked meteorites (lines 102–103), a question finally addressed in our high-pressure deformation experiments. Furthermore, the more dominant role of the NPL in both kinetics and rheology, as found in our experiments, is also consistent with meteorite data (lines 102–105). In the context of the unique and valuable similarities between conditions in shocked meteorites and the deep slab, we have kept this sentence and the information remains unchanged.

4. NPL and STL Angles (Line 100): The statement regarding the orientation of NPL at high angles to the deformation axis requires statistical backing. A figure presenting the statistical distribution of NPL and STL angles would strengthen this claim and improve the manuscript's clarity.

We agree with the reviewer’s suggestion and add a figure showing the distribution of STL and NPL including NSBs angles with respect to the deformational axis (Extended Data Fig.10).

5. Latent Heat Comparison (Lines 193-201): The authors discuss latent heat and compare germanate to magnesium silicate systems, but the assertion that latent heat release in germanate systems is more significant lacks appropriate citations (Line 196). Additionally, the connection between the discovery of nanocrystalline fine grains in germanate systems, the orientation of faults at 45 degrees relative to the sigma1 direction, and greater latent heat release in germanates is unclear. This section could benefit from clearer articulation and reference support to bolster the argument.

We agree that this section was unclear. We originally intended to compare our results with previous germanate studies to emphasize the ubiquity of the instability mechanism over a large pressure range, extending beyond the BPT condition. We revised the manuscript to clarify our aims (lines 209–226) and added a reference on the latent heat release in lines 225 (originally line 196).

6. Geological Implications (Lines 239-255): The application of the experimental results to real geological examples could be expanded. Deep earthquakes occur in only a limited number of locations globally, and thus, a broader synthesis of observations would improve the implications section. I recommend including a table or graph summarizing the findings, their implications, and their applications to better illustrate how the experimental results relate to deep-earth

phenomena.

Thank you for the suggestion regarding the summary diagram. As the reviewer pointed out, previous studies have clearly shown that the locations of deep earthquakes are limited and highly sensitive to temperature conditions, with the occurrence rate peaking at around 600 km depth. While our study represents a breakthrough by reporting transformation-induced adiabatic instability and its transition to stable sliding (rheological weakening) close to these peak conditions, our results do not rule out the possibility of other mechanisms such as dehydration embrittlement. Furthermore, it remains unclear what causes the peak in deep seismicity, which may depend on slab conditions such as stress amplitudes and hydration levels, and/or pressure-dependent material properties such as transformation, dehydration, and frictional behaviors. At this stage, further systematic studies, particularly across a broader range of pressure and water conditions, are crucial for understanding the overall characteristics of deep seismicity. Therefore, although it would be ideal to include a figure summarizing our findings and their implications, as the reviewer suggested, it is better to wait until further systematic studies have been carried out.

7. Re-emphasizing Mohiuddin et al. (2020): While the authors reference Mohiuddin et al. (2020), the significance of this prior study could be highlighted more. I understand the authors did not intend to downplay its contribution, but after reading this manuscript, I was left with the impression that the idea of grain-size reduction-induced weakening for deep-focus earthquakes was novel to this study. I recommend a revision that better acknowledges the importance of this prior work, ensuring that the manuscript appropriately credits the foundation laid by Mohiuddin et al. (2020).

We agree that the achievement of Mohiuddin et al. (2020) should be emphasized more as pioneering work. They demonstrated that fine-grained ringwoodite deforms by grain-size sensitive creep, which leads to slab weakening. However, they just observed the presence of fine-grained ringwoodite in their recovered sample, and the details of transformation and deformation microstructures were poorly understood. We newly observed that the stress-induced formation of NPL is a key triggering superplasticity, providing evidence that fills the gap in the limitations of their study. As noted at the beginning, our focus is on the mechanism of deep-focus earthquakes induced by the ol-rwd transformation, which could not be addressed in Mohiuddin et al. Therefore, the idea of grain-size reduction-induced weakening for deep-focus earthquakes was first demonstrated in this study. Our study provides insights into the connection between deep-focus earthquakes and the slab weakening mechanism studied by Mohiuddin et al. (2020). We have incorporated these points in lines 143–149 and 327–331 of the revised manuscript.

In summary, the manuscript presents interesting and robust experimental results with significant

implications for the study of slab dynamics in the MTZ. However, it would benefit from revisions to clarify and strengthen the discussion, particularly regarding its novelty, the application of results to composite slabs, and the connection to prior work.

We appreciate your constructive feedback once again, most of which was missing from our perspective. We have revised our manuscript based on your comments, which have significantly improved our paper.

Reviewer #2 (Remarks to the Author):

The authors present an experimental work on the long-standing issue of the origin for deep earthquakes occuring within the transition zone, that may be caused by transformation of metastable olivine to its high-pressure polymorph ringwoodite. They use high pressure large-volume experiments, coupled with ex-situ characterization of the samples microstructures, and with in-situ x-ray diffraction and acoustic emission measurements which provide insights into the stresses and brittle character of the deformation. From these results they calculate frictional properties of nano-polycristalline ringwoodite shear bands and investigate their potential to become « seismic » due to latent heat release and frictional shear heating.

This topic has been addressed before using similar coupled methods - a number reference works are duly cited in the introduction.

However, this study stands out as an extensive experimental work on the system using the actual olivine composition in the mantle - as opposed to previous ones at lower pressures or using analogs. The deformation experiments by themselves are extremely challenging to carry out at these pressures and the dataset seems pretty unique. The study also includes documentation of microstructural relationships for different shear bands types, using microscopy, on samples deformed at these pressures of 20 GPa, which has been seldom made I beleive.

The extrapolation to nature is more speculative and contains some weaknesses (comment (3)). The publication should be of great interest to communities working on the earth and planetary interiors from mineralogists to modelers and seismologists. The manuscript is well written and well organized. The figures are informative and clear.

I would recommend publication of this work, with modifications. Here are some reservations or questions on this manuscript, (3) and (2) being the most important ones :

We sincerely appreciate your understanding and critical comments on our manuscript, which were extremely important and helpful in improving our discussions. We have revised our manuscript taking your comments into consideration. Some questions have been separated and answered as necessary.

1) The presentation of the coexistence of weak zones and strongly stressed zones in the MTZ as

a « paradox » is simplistic to me (abstrast, beginning of the introduction). Line 49 on the stagnation of slabs : how about the influence of density contrasts ?

We partly agree with the reviewer's comments regarding the term "simplistic". We have removed the sentences on paradoxical coexistence, and instead mentioned that they are both paradoxical issues from a mineral physics perspective (shear instability at MTZ pressures and rheologcal weakening at cold slab temperatures) in the abstract and at the beginning of the introduction. As the reviewer pointed out, the density contrast is an important factor in slab stagnation. Several previous studies involving slab dynamics simulation have indicated that weak slab rheology (i.e., relatively low yield strength) is also necessary to explain the slab behaviors. We have addressed both points in the revised manuscript (lines 52–55).

2) Lines 127-131 (and lines 210-211): the flow law calculation using diffusivity remains unclear. It seems the flow laws for diffusion used here have been established at higher grain sizes (>micron) and higher temperatures (1400 to 1600°C) (reference 16). Can these be extrapolated down to 20 nm grain sizes and 1000°C (figure 3b) ? Or even farther, to the conditions of the lowest temperature runs (fig. 3c,d) ?

As the reviewer pointed out, Shimojuku et al. (2009) [ref17] determined the diffusivity of ringwoodite under normal mantle conditions. To extrapolate these data to slab conditions with lower temperatures and finer grain sizes, accurate estimates of the temperature dependence of both volume diffusion and grain-boundary diffusion coefficients are essential. They provided these valuable data. Therefore, in principle, we can estimate the effective diffusion coefficient for varying grain sizes at different temperatures. This approach quantitatively describes the enhancement of diffusivity in finer grain size at lower temperatures due to the contribution of grain-boundary diffusion with a small activation enthalpy. We believe that, at this stage, this is the best method to infer the strength of fine-grained ringwoodite, as previous studies have done (e.g., Kubo et al., 2009 [ref2]; Mohiuddin et al., 2020 [ref10]).

With these uncertainties, it is difficult to trust that the calculated (huge) difference in stress caused by variations of grain sizes from 20 nm to 27 nm, is significant (line 131). Moreover, the grain sizes range in the bands, is probably wider than 20 to 27 nm in the actual sample.

As the reviewer suggested, the difference in stress with grain size is significant, but this is a general characteristic of diffusion creep. In the case of Coble creep (i.e., grain-boundary diffusion-dominated creep in fine-grained materials), the flow stress increases with grain size to the power of three. Therefore, the increase in grain size from 20 nm to 27 nm corresponds to a ~2.5-fold increase in stress, which is roughly consistent with the stress-strain curve shown in Fig. 3b (from ~1.5 GPa to ~4.0 GPa).

Regarding the latter issue, we understand the concern raised by the reviewer. Strictly speaking, the

grain size of ringwoodite in the recovered sample should be 27 nm to exactly match the observed stress. However, the grain size of polycrystals is generally not constant and follows distribution. Because it was difficult to measure the grain size distribution due to the narrow region observed in the TEM image, we do not intend to present a quantitative discussion here. Instead, we emphasize that the observed mechanical behaviors, such as constant strength during temperature ramping and hardening at a constant temperature, strongly suggest superplasticity in ringwoodite. This is not a new observation but has been previously reported in Mohiuddin et al. (2020) [ref10], as mentioned in the manuscript. Our observations advance their study by clarifying the transformation microstructures and further exploring the localization and shear instability in relation to deep earthquakes. We have slightly revised the manuscript to address the difficulty of providing a quantitative discussion (lines 132–149).

Finally, can this flow be called superplastic (line 126 - a reference would be welcome, and lines 136, 211) ? What is the relationship with possible superplasticity with grain boundary gliding or sliding ?

As the reviewer suggested, superplasticity is characterized by large strains associated with significant grain-boundary sliding (cf. Karato 2008, "Deformation of Earth Materials"). Diffusion creep at large strains involves grain-boundary sliding, and the terms superplasticity and diffusion creep have often been used to describe essentially the same phenomenon in the literature. However, we did not have clear evidence for grain-boundary sliding, such as four-grain corner sand grain switching events, in our samples. Therefore, we will refrain from using the term 'superplasticity' (or 'super plastic flow') and instead use 'diffusion creep' and/or 'grain-size sensitive creep' in our paper. This does not affect the significance of our study, as the key process for reaction-induced rheological weakening and shear localization is that the rate of deformation is sensitive to grain size.

3) I believe heat dissipation cannot be overlooked and should be addressed in the main text (ie. the lines 533-537 should be with Figure 4 and lines 221-237). To me, a proper discussion between timescales for heat dissipation, that occurs on the microsecond, and the timescales involved for the processes that produce shear strain, is needed. This affects the overall significance of figure 4 and the associated discussion.

We have briefly addressed the timescales for latent heat dissipation in the revised manuscript (lines 253–258). Although this timescale is not directly linked to the overall process of instability, we note it in the context of introducing the concept of an "instantaneous" frictional coefficient. This concept is relevant to the subsequent stage of frictional heating, in which shear localization–induced by latent heat– further amplifies heating through friction.

While we acknowledge the reviewer's emphasis on the importance of timescales, we find that a detailed discussion of the full evolutionary pathway to instability, along with its associated timescales,

is beyond the scope of the current study. Nevertheless, we have identified and discussed several key steps leading to transformation-induced thermal instability: 1) the formation of NPL, 2) weakening assisted by latent heat that results in the formation of NSBs, and 3) the onset of frictional heating capable of reaching seismic strain rates via grain-size sensitive creep.

These processes are now discussed semi-quantitatively in the revised text, with consideration of both spatial and temporal scales relevant to laboratory (lines 253–258 and 274–283) and deep slab earthquakes (lines 286–326). We believe this expanded discussion supports our conclusion that the experimental observations provide a plausible mechanism for deep earthquakes occurring at depths of ~600 km.

In the same line the extrapolation of the experimental results to earthquakes is not so strong here, based on a simple ratio D/h ; that does take into account possible changes in the instabilities propagation mechanisms, from the sample scale, to the fault/earthquake scale.

Although we are not confident that we have fully understood the reviewer's comments, we agree that extrapolating to the earthquake scale is not always straightforward, as the instability process may be influenced by scale effects. In fact, little is known about the sliding conditions for both deep earthquakes and lab earthquakes. Among these factors, we believe that the *D/h* ratio is a simple and useful parameter across a wide range of scales.

In the lab scales, Wang et al. (2017) [ref 7] suggested that the fault formation involving NSBs occurs through a propagation process where the initiation and termination points of individual segments connects, based on the microstructural observations and the spatial distribution of fault planes and AE events. We consider that a similar mechanism may be present, given the resemblance between our observations of NSB behavior at 20 GPa and those from their lower-pressure experiments using analogue materials. Furthermore, under conditions of large strain, fault thickness remains on the order of 10^1 times the grain sizes (cf. Vardoulakis and Aifantis, 1991 Acta Mech.; Wang et al., 2017), while slip displacement *D* increases. This suggests that in larger-scale faults, the *D/h* ratio increases, leading to further localization. Similar phenomena can be observed in natural faults, where the gouge is typically composed of micron-scale fine-grained material and remains very thin. Although information on deep earthquakes is limited, the largest *D/h* ratio of ~4000 has been reported in a Bolivian deep earthquake, as mentioned in the original manuscript, where *D* and *h* values were estimated to be 15.6 m and 3.7 mm, respectively. Therefore, the scaling from the small *D/h* ratio at high stresses in the lab to the larger *D/h* ratio under lower stress conditions in natural fault seems to be established, with frictional heating playing a key in instability across a wide range of scales.

Fault linkage and self-organization processes, similar to those observed in brittle failure, may introduce additional complexity at larger scales. However, capturing such processes experimentally remains challenging, even at lower pressures, and developing a comprehensive model is beyond the

scope of this study.

On this issue, we did not provide details but added several sentences referencing the results of a previous study regarding the larger *D/h* value at larger scales (lines 267–270).

4) Stress in olivine are measured through diffraction lines 130 and 021, which usually are on the high-end of the stresses calculated from the different crystallographic planes in olivine. The stress in the aggregate may be lower than that (e.g. Nishihara et al, 2008 PEPI, works by Burnley and collaborators showing the need for EVPSC models (Burnley et al., 2019 AmMin.), ...). What would be the consequences for the calculations ?

As the reviewer noted, previous studies indicated that the olivine stress measured through diffraction lines 130 and 021 tends to show values up to ~20% higher than the average values (which include other diffraction lines such as 131, 112, and 101) in the similar low-temperature plasticity regime (cf. Mei et al., 2010 [ref47]; Long et al., 2011 [ref48]). However, we could only use the diffraction lines 130 and 021 because the other lines were overwrapped by diffraction from other phases (cf. Extended Data Fig. 6). This resulted in an overestimation of the equivalent shear stress by ~0.5-0.6 GPa compared to the average value. On the other hand, we measured the ringwoodite stress using diffraction lines 400 and 440, which correspond to low- and high-end values, respectively (Kawazoe et al., 2016 [ref49]; Mohiuddin et al., 2020 [ref10]), and thus we regard their average as a reasonable approximation of the representative stress value. Although this may slightly affect the discussion of the relative strength of these phases in Fig. 3 and shear heating in Fig. 4, the effect is considered minor, as the degree of the overestimation is close to the error in the stress calculation. We briefly mentioned this issue in the revised manuscript (lines 558–565).

5) The number of AE events recorded (Extended table 1) is very low, e.g. relative to studies like Schubnel et al, 2013 [ref6]. On the other hand, Schubnel et al. did not observe stress drops from the diffraction patterns. Could these differences be due to different mechanisms, or different experimental strategies?

These differences could be derived from both. The pressure differs significantly between these studies (~2–5 GPa and ~20 GPa), which may have a substantial impact on the nature of frictional sliding, leading to the difference in the number of AE events and degree of stress drops. However, the number of AE events may also be influenced by the differences in transducers and amplifiers. The PZT transducers with similar resonant frequencies around 2 MHz were used in both studies, but the diameter was larger, and the signal was amplified to higher levels (45–60 dB, compared to 20 dB in this study) in Schubnel et al. (2013), making it possible to collect a larger number of AE events.

On the other hand, the degree of stress drop may depend on the strength of the parental olivine. Schubnel et al. (2013) reported a smaller stress drop of ~100 MPa when the stress of Ge-olivine was

2.7 GPa, while our data showed the larger stress drop of ~1 GPa with a higher olivine stress of 5-6 GPa. The strength of (metastable) olivine is relatively high at mantle zone pressures, which may lead to the large stress drop observed in our study. Also, the release of latent heat is more significant in magnesium silicate, which may contribute to the occurrence of the larger stress drop. From the methodological perspective, the time resolution of stress measurement was slightly poorer in their study, which may have resulted in failing to detect the stress drop.

Furthermore, the development of shear instability depends on the stiffness of the surroundings (cf. Karato, 2008). Although a quantitative discussion is difficult at this time, differences in apparatuses (D-DIA vs D-111) and/or cell assemblies between the two studies may influence the magnitude of instability.

Thus, although the reviewer's comment may be essential for the understanding of the pressure effects on the faulting, its interpretations are still unclear due to the differences in experimental method with pressures. Among them, the large repetitive stress drop observed in this study is valuable for discussion because no previous studies are addressed, which is likely due to the pressure effects. We added some remarks on this point in the revised manuscript (lines 220–226).

6) lines 213-220 : can this latent heat release hypothesis really be applied to a couple of lenses of ringwoodite, such as those for run Oldt51 (last extended figure), that do not form a continuous band ?

Our hypothesis is only true the region where the transformation initiated. The samples which experienced instability have conjugated ringwoodite lamellae (NSBs) shown in Figs. 2c and 2d and deformation lamellae with little or no ringwoodite (Extended Data Fig.12) at the same time. Although that may be caused by the subtle temperature and/or stress-strain distributions in the sample, once the transformation occurred, the instability initiated from the region and developed following our model. On the other hand, we require the transformation (and thermal runaway) only near the NSBs and/or "fault", so we consider it is no matter whether the regions without transformation remain which do not contribute to the fault formation.

7) On the stress measurement geometry : It would be useful to add arrows showing where the maximum stress is on the 2D diffraction pattern figure 6a. The authors made a correction for absorption of the sample width change, with increasing strain. Making a correction for the sintered diamond anvil absorption may be more important, since it seems to affect the diffraction intensities with respect to the azimuth angle. Or is the olivine texture, relative to the maximum stress, unusual ?

We added the direction of maximum stress and the anvil gap to the 2D XRD pattern in Extended Data Fig.6a. We estimated the transformed fraction from the diffraction intensity integrated along

azimuthal angle. To account for the increase in sample volume radiated by X-rays with increasing sample strain (width), we applied a correction. As the reviewer pointed out, the diffraction intensity obtained through the anvil gap tends to be larger than that through sintered diamond anvil. However, this heterogeneous intensity distribution remained constant over time, and therefore did not affect our estimate of the transformed fraction and the stresses.

While we acknowledge that our understanding of the reviewer's last question concerning the olivine texture might be incomplete, the intensity distribution can be influenced not only by the anvil gap, but also by the olivine texture itself. This latter effect, however, was not clearly discernible in our data, and we did not consider it.

Line by line comments:

Line 53 : I do not understand; should 'cause' be replaced by 'undergo' ?

Line 124 : « the stress of ringwoodite remained »

We revised these mistakes.

Line 138 : low temperature plasticity could be more appropriate than « Peierls ». The activation volume chosen is on the very high-end side.

We revised it to "low-temperature plasticity" (line 152). Our results indicate a significantly high strength for olivine under our pressure conditions, implying a large pressure dependence (i.e., high activation volume) in the low-temperature plasticity regime. This strong pressure dependence has been largely overlooked in previous studies, likely due to pressure limitations up to around 9 GPa (e.g. Mei et al., 2010 [ref47]; Long et al., 2011 [ref48]; Proietti et al., 2016 PEPI). However, the upper range of previously published activation volume values can account for the olivine strength observed in our experiments. Furthermore, a recent study conducted under shallower MTZ conditions also supports this finding of a large pressure dependence in olivine strength (Ohuchi et al., 2022 [ref8]).

Line 209-210: not so easy to understand, maybe try reworking the sentences.

Line 214 « at the instant when » english can probably be improved here

Line 243 : « location of foci of deep ...» : location of deep earthquakes ?

We revised and improved these sentences.

<u>Reviewer #3 (Remarks to the Author):</u>

The formation mechanism of deep-focus earthquakes has been a long-standing question for rock physics and seismology communities for several decades. This work used only olivine as a starting material and focused on the in-situ observations for the deformation and phase transformation of olivine to ringwoodite at pressure and temperature conditions corresponding to the bottom of MTZ.

The pressure-induced formation of nanoshear bands (NSBs) has been reported from previous reports (Burnley et al. 1991; Schubel et al. 2013; Wang er al. 2017) using Mg_2GeO_4 and hazburgite-like composition. The experimental method used synchrotron X-ray together with acoustic emission for deformation and rupture detection have also been demonstrated previously for deep-focus earthquake studies. This work is similar as report from Burnley et al. (1991), Schubel et al. 2013 and Wang er al. 2017 but provides with slightly different analyses (Fig. 2) in SEM and TEM from their quenched products. What makes the contribution different from others? The authors used mantle dominate olivine at P-T conditions close to subducted cold slabs. They clearly demonstrate the formation of NSBs associated with the uniaxial compression direction and explain the formation of conjugated faulting zone. Importantly, they suggest the appearance (nucleation) of ringwoodite plays a key role for the formation of weak plane and thus causes the shear instability. The shear strain rate with respect to shear stress shows reasonable explanations for the superplastic flow in ringwoodite that may be related to subducted slabs.

We appreciate your constructive review and comments. Prior to addressing your detailed comments below, we would like to respond to your key concerns regarding the perceived similarity of this work to previous studies and the novelty of our contribution.

We acknowledge that this long-standing question on deep earthquakes has been addressed in numerous high-profile publications, including many in Nature and Science, underscoring the importance of this topic across various research fields. To achieve a definitive resolution, pressure must be considered a paramount factor, given the strong pressure dependence of frictional strength (i.e., Byerlee's law). The critical question to be resolved is why deep earthquakes occur at pressures significantly exceeding the brittle-plastic transition (BPT), typically around 2-3 GPa. Furthermore, the analogue materials employed in previous studies exhibit distinct chemical properties and transformational behavior compared to mantle silicates (e.g., bonding, structures of high-pressure phases, and latent heats). From this perspective, our work, conducted at mantle transition zone (MTZ) pressures, represents a significant departure from previous studies performed around BPT pressures (e.g., Burnley et al., 1991; Schubnel et al., 2013; Wang et al., 2017 [refs 5–7]). Notably, even an author of these previous studies acknowledges this distinction. Ultimately, conclusive results can only be achieved through direct experimentation at MTZ pressures of ~20 GPa, the region where deep earthquakes occur most frequently.

We would like to emphasize that our results are also crucial outcomes that bridge the gap between "analogue" and "real" conditions. To achieve this, we further developed methods for detecting shear instability, such as synchrotron X-rays and AE, which were employed in the previous study (Schubnel et al., 2013). Applying these techniques to MTZ pressures was exceptionally challenging, requiring ~10 years, including the development of a novel deformation apparatus (D-111).

Consequently, by conducting experiments using mantle olivine at MTZ pressures, we have been

able to directly constrain both the detailed process triggering deep earthquakes and the temperature conditions that delineate the distinct slab behaviors of deep seismicity and bulk weakening. Therefore, our findings represent a significant advancement in solid earth science compared to earlier work.

Besides, there are some minor questions needed to be answered.

Line 94-97 describes the interesting orientation of rwd grown under high P-T in the Fig. 1 with fine grains in central layer and outside with elongated and roughly perpendicular oriented grains. Any more detailed photos to show this?

Unfortunately, we do not have more detailed images of this microstructure. However, we have observed similar textures in L6 chondrite, as cited in the manuscript. We have also observed similar textures during the olivine-spinel transformation in fayalite, but this observation is from unpublished work.

Line 129 "…hardening behavior at constant temperature can be explained by the changes in grain size from 50 nm to 200 nm, and from 20 nm to 27 nm, respectively…" seems confusion. The constant temperatures shown in Fig 2a and 2b did not clearly exhibit stress hardening behavior, especially for rwd440. Furthermore, what's the explanation for the drop of strength on rwd400 at strain rate of 34-45%?

First, we would like to clarify that this issue pertains to Fig. 3a and 3b, not Fig. 2a and 2b. In Fig. 3, we observed hardening behavior in two distinct ways: constant stress despite temperature ramping (Fig. 3a), and stress increase at constant temperature (Fig. 3b). Therefore, the absence of hardening in the stress data for rwd440, as shown in Fig. 3a, does not pose a problem.

Regarding the drop in strength shown in Fig. 3b, the underlying reasons remain unclear. One possible explanation may involve heterogeneous distributions of rwd grain size and stress within the sample during transformation and deformation. Specifically, newly formed lamellae with finer grains may have continuously developed. Although the stress was averaged along the X-ray path, these heterogeneities could potentially be detected because the radiated area shifted slightly within the sample during the deformation experiment. Additionally, we acknowledge that the uncertainty in stress measurement is not small, particularly when the transformed fraction is small, due to the weak intensity of diffraction peak.

We have revised the sentence on lines 130–143 to clarify our original intent, which was not to demonstrate precise grain growth from 20 to 27 nm, but rather to illustrate the general trend of stress hardening associated with grain growth.

Line 138 state the high dislocation density in olive. Can the authors elaborate to explain this explicitly? It's a bit difficult to understand in Fig. 2b.

TEM observations revealed that olivine is heavily strained particularly in the sample deformed less than ~1000°C (Figs. 2b, 2c, and extended Fig. 12b). However, as suggested by the reviewer, the deformation texture's clarity is limited due to the relatively thick FIB foil (~100nm) and the high density of tangled dislocations. This limitation is noted in the figure legend of Fig.2. Consequently, we have revised line 151 (originally line 138) to reflect the observable evidence, replacing "high dislocation density" with "heavily strained".

Why rwd440 shows in Fig 3a but rwd400 shows in Fig 3b?

First, to clarify the methodology, rwd stress could be measured from both the 400 and 440 reflections in Fig. 3a, but only from the 400 in Fig. 3b. This difference stems from the experimental setup: Fig. 3b (run oldt23) employed a slotted WC anvil, which restricts XRD data collection to 2θ below ~6°. Conversely, Fig. 3a (run oldt12) utilized a sintered diamond anvil, permitting data collection up to 2θ ~10°, and thus enabling the observation of the rwd440 diffraction.

Our initial concern was that displaying labels for both rwd400 and 440 in the spatially constrained Fig. 3a might lead to visual clutter. Consequently, we initially placed the rwd440 label (orange) in Fig. 3a and rwd400 label (brown) in Fig. 3b. However, this approach inadvertently caused confusion. To rectify this, we have now included captions for both rwd{400} and rwd{440} directly within Fig. 3a.

Line 210 shows the definition of superplastic flow (i.e. diffusion creep) that needs to be defined in early paragraph (Line 126).

We agree with the reviewer's comment. However, based on feedback from another reviewer, we considered it more appropriate to avoid the use of the term "superplasticity" and instead directly refer to "grain-size sensitive creep" and "diffusion creep". Accordingly, we have revised the text to reflect this change (e.g., lines131–132, 235).

Line 222, please explain the velocity-strengthening behavior.

We added a simple explanation and a reference (ref. 21) (lines 251–252).

NCOMMS-24-66673-T

Title: The olivine-ringwoodite transformation triggers deep slab seismicity and rheological weakening

Authors: Rikuto Honda et al.

Late Update: January 27th, 2026

Responses to Reviewers' comments

(Authors' responses and the line numbers in annotated revised manuscript are shown in red.)

Reviewer #3 (Remarks to the Author):

This work emphasizes their findings on the formation of stress-induced NPL that may cause grain-size sensitive creep and thus trigger the deep-focus earthquake at low temperatures. The authors have answered most of the questions raised by the reviewers and provided an updated revision for better presentation of their work. I recommend publishing this work. However, there are still two minor issues to be resolved.

We appreciate the reviewer's comments. We consider these two points to be important for further improving the clarity and quality of the manuscript.

Reviewer 1 asked the authors to provide a figure presenting the statistical distribution of NPL and STL angles to clarify the claim which I also agree. However, the newly added Fig. 10 did not show the errors in this plot that may weaken the argument.

Following the reviewer's suggestion, we have added errors (binomial standard error) to Extended Data Fig. 10 (lines 810–811).

In Fig.4a, the shear heating temperature plot is helpful for showing the thermal instability but it is difficult to read from the plot. For example, Line 263-264 states D/h ~1.5 …is expected to increase the temperature by ~750–940°C. However, it is hard to read this temperature from Fig. 4a. Furthermore, the TZ temperatures are expected in the range of 600-2000°C. It is thus suggested to show the temperature scale not exceeding 3000 (or 2500) °C to allow readers to read the meaningful shear heating temperatures.

We agree with the reviewer's suggestion. For improved readability, we have revised the maximum value of the vertical axis in Fig. 4a to 2,500°C.

Reviewer #4 (Remarks to the Author):

Review of manuscript "The olivine-ringwoodite transformation triggers deep slab seismicity and rheological weakening" by Honda et al., submitted to Nature Communications.

This study reports novel experimental measurements that characterize the coupling between the

olivine-to-ringwoodite phase transformation and deformation processes, highlighting their significance for understanding deep-focus seismicity and mechanical weakening of subducting slabs under mantle transition zone conditions. To accomplish this goal, the authors implemented a comprehensive experimental approach that integrates high-pressure large-volume deformation experiments with in situ X-ray diffraction and imaging to quantify stress–strain behaviour, acoustic emission monitoring, and detailed ex situ microscopic examination of the resulting microstructures. The key finding is that stress-induced formation of nanopolycrystalline ringwoodite initiates deep-focus seismicity under low-temperature conditions, while the subsequent bulk phase transformation results in homogeneous rheological weakening at higher temperatures. This conclusion is not entirely new, as it has been proposed in several previous studies employing essentially similar methods on analogue materials. However, the significance of the present work lies in the fact that the measurements were performed directly on Mg_2SiO_4 olivine under mantle transition zone P-T conditions—an important experimental achievement. The study therefore provides compelling confirmation that the mechanisms identified in analogue systems are also operative in natural compositions. This in itself justifies the publication of this work, which will be of interest to a wide audience within the deep earth scientific community.

I did not evaluate the initial version of the manuscript, but I carefully reviewed the modified version and the response to the comments of the previous reviewers. I found the conclusions to be robust and well supported by the in situ measurements and the post-mortem microscopic analyses of the samples. The methods are well developed, and the article is clearly and effectively illustrated (with useful extended data figures), in part thanks to the reviewers' constructive feedback.

Of course, extrapolation to natural conditions is complex given the mineral heterogeneity of the plates, their stress state, grain sizes, the role of heat dissipation, etc…, and may be open to debate. However, these points are beyond the scope of this study, as the authors point out.

My only reservation concerns the treatment of uncertainties in the sections on the interpretation of the local temperature increase due to latent heat of transformation, the initial grain size of ringwoodite in the NSBs, as well as friction heating and the rate of shear deformation on the fault plane. Including a more detailed discussion that accounts for the uncertainties in the different parameters would provide valuable insight into the sensitivity of the conclusions to these factors.

Apart from this comment, this revised version fully meets the scientific and presentation standards required for publication in Nature Communications with only minor adjustments.

We sincerely appreciate the reviewer's thorough review and valuable comments. We respond below to the specific concern identified above (highlighted in bold).

The uncertainties raised by the reviewer are indeed important for interpreting Fig. 4. We

acknowledge that these parameters involve inherent uncertainties, most of which have already been discussed in the manuscript. Among the parameters highlighted, grain size, stress, and D/h value are the most critical, and their uncertainties are explicitly treated as variables in Fig. 4. Below, we respond to each point raised by the reviewer.

Local temperature increase due to latent heat of transformation:

The magnitude of the temperature rise due to latent heat (240–270°C, lines 629–632) depends on factors such as volume change and the temperature dependence of heat capacity in addition to the pressure and temperature (Eqs. 6 and 7 in methods). These parameters were not measured in this study but were instead taken from previous studies (Kojitani et al., 2012 ref 54; Liu and Li, 2006 ref 55; Nishihara et al., 2004 ref 56). Even when accounting for the uncertainty reported in these studies, the resulting error in the temperature rise is limited to ~20–30°C for the seismic temperature both in laboratory and slab. This does not affect our conclusion that latent heat induced temperature increase can trigger shear localization (lines 237–239). We have slightly revised the manuscript to note that these uncertainties were considered (lines 629–632 in methods).

The initial grain size of ringwoodite in the NSBs:

We considered both the Avrami length derived from transformation kinetics and the minimum grain size constrained by the energy balance for nucleation as estimates of the initial grain size of ringwoodite (lines from 643 in methods). These considerations indicate that the latter dominates (4.5–6.7 nm) under both lab and slab conditions, which is almost consistent with the observed grain size (~10 nm) considering grain growth after the transformation. While the shear heating in natural deep earthquakes that may become much larger than in our experiments (lines 306–308), we explore a plausible range of grain sizes from 10 to 100 nm in Fig. 4 (lines 474–475). Smaller grain sizes weaken NBSs increasing shear strain rates, whereas larger grain sizes harden NBSs and enhance shear heating. Thus, even accounting for this uncertainty, either seismic strain rates or frictional melting are expected for realistic D/h values, indicating that our conclusions are robust.

Friction heating and the rate of shear deformation on the fault plane:

Frictional heating depends on stress and the D/h ratio (Eq. 10). Smaller D/h values require higher stresses to generate sufficient heat to achieve seismic strain rates or frictional melting. Because these parameters are correlated and vary over wide ranges owing to the diversity of slip scales from laboratory experiments to slab earthquakes, we treated them as variables in Fig. 4.

In laboratory experiments, the D/h ratio is necessarily very small, and instability requires stresses on the order of GPa, as demonstrated in our experiments. In contrast, for natural deep earthquakes, the D/h ratio can be as large as several thousand (e.g., Kanamori et al., 1998, refs. 33 and 34), such that

stresses of only several MPa are sufficient. This stress range is broadly consistent with plausible slab conditions, ranging from several MPa to several GPa (refs 28–31). The lower bound of this range is constrained by observed stress drops (e.g., Liu et al. 2020, ref 31), which provide a minimum estimate of slab stress.

We therefore conclude that Fig. 4 captures these relationships and demonstrates that seismic strain rates or frictional melting can be achieved within realistic ranges of these parameters.

Thus, the uncertainties in the key parameters raised by the reviewer (the amount of latent heat release, initial grain size, stress, and *D*/*h* ratio) are already incorporated in Fig. 4 by exploring a range of plausible values, and their impact on our conclusions is minor. We agree that the manuscript did not explicitly describe how these uncertainties were treated. We have therefore revised the text to provide a more detailed explanation (lines 274-280 and 298–318).

Reviewer #5 (Remarks to the Author):

Summary

The purpose of this article is to share recent findings on the nature of rheological weakening, shear instability, and the effects of transformation of metastable olivine to ringwoodite within a subducting slab in the mantle transition zone, as well as to explore the contribution of grain-size sensitive creep to these large-scale behaviors. The details of these processes have not previously been explored or characterized in detail experimentally. The authors aim to do this using high-pressure, high-temperature in-situ deformation experiments and acoustic emissions measurements. Samples are compressed up to 20 GPa at room temperature, and then uniaxially deformed between 570 – 1,330 °C with a constant anvil displacement rate using a D-111 type deformation apparatus. Major findings include the formation of high-pressure phases nano-polycrystalline lamellae (NPL), single crystalline topotactic lamellae (STL), and nanoshear bands (NSBs), all of which are thinly nucleated ringwoodite phases dispersed in certain areas of the sample or throughout the sample, respectively. These phases are key in rheological weakening via grain-size sensitive creep. Acoustic emission (AE) measurements and XRD were used to further characterize the coupling of phase transformation and deformation by seismicity. This work supports previous work suggesting that the aforementioned microstructural processes lead to slab weakening and potentially deep earthquakes.

We sincerely thank the reviewer for the clearly organized and constructive review. For comments that include suggestions or questions, we have highlighted them in bold and provided our corresponding responses below.

Strengths

- The work is novel in its effort to experimentally explore the coupling of deep seismicity and the

high-pressure phase transformation of olivine. The experiments carried out are detailed. The results are well presented with clear diagrams.

- Care is taken to distinguish the results of deformation by temperature and pressure, because some of the processes of interest are kinetically controlled.
- The practice of in-situ deformation experiments with X-ray synchrotron presents trustworthy quantitative results, further bolstered by the cross-correlation of the XRD and AE results.
- The development of a new KMA-type acoustic emission measurement system may be useful for future AE studies.
- **Figure 1A clearly portrays the NPL and STL deformation features. The figure would benefit from having NSBs on the photo as well, for comparison.**
- Authors present a strong qualitative analysis of NPL's contribution to grain-size sensitive creep, including Figure 3, which highly motivates future quantitative studies on the topic.

Thank you for your accurate review regarding the strengths of our study. We would like to provide our response to the suggestion included in the fifth comment. We defined the term of "NSBs" as lamellae inducing localized deformation (lines 112–114). However, any localizations did not occur in the sample at high temperature shown in Fig. 1 and NSBs were absent in the sample. As this relates to the reviewer's the next comment as well, we have revised the manuscript to clarify these observational facts and their roles more explicitly (lines 107 and 113–114).

Challenges

- **The functional distinction between nanoshear bands (NSBs) and the lamellae (single-crystalline topotactic lamellae (STL) and nano-polycrystalline lamellae (NPL)) is a bit unclear. I suggest providing further clarification in the section titled "Formation of nanoshear bands at medium T".**

We have clarified that only (the initially formed) thin NPL act as NSBs that induce localized deformation, and that STL do not contribute to this process (lines 113–114). We also emphasized that neither STL nor NPL at higher temperatures produce localized deformation (line 107).

- **The values in Extended Data Table 1 would benefit from adding uncertainties to allow readers to evaluate data spread.**

Information of uncertainties were added for all parameters for which they could be reasonably estimated. We also added a statement regarding the uncertainties of pressure and temperature to the caption of Extended Data Table 1. In addition, we found a partial transcription error in the pressure values in Extended Data Fig. 4, which has now been corrected (this was only a display error and does not affect any other contents).

- **The work addresses the question of deep seismicity in the metastable olivine wedge, which is relatively cold compared to the surrounding mantle and outer slab. Some experiments explored these lower temperatures, but no ringwoodite or acoustic emissions were formed in these runs. Latent heat was suggested as a source of the additional heat needed to facilitate these reactions (line 297), but without extrapolating, this result is worrisome.**

As noted by the reviewer, no transformation occurred and no acoustic emissions were detected below 760°C in our experiments, despite deep seismicity being expected at such low temperatures. We attribute this to kinetic limitation. Although the transformation does not proceed within the laboratory timescales at these temperatures, previous kinetic study indicate that it can occur down to ~550°C over geological timescales in the metastable olivine wedge. As discussed in lines 281–287 (originally around line 297), the olivine–ringwoodite transformation triggers thermal localization through latent heat release. The initiation of the transformation is governed by time-dependent kinetics, and once initiated, it is further accelerated by positive feedback from latent heat release. Thus, as noted by reviewer, although latent heat provides an additional heat source that facilitates the transformation, it can not be used to lower the initiation temperature. Instead, the much longer timescales of subducting slabs allow the initiation temperature to be effectively lowered, consistent with conditions in deep seismicity regions.

- **There did not appear to be consideration for other kinetic mechanisms besides the major grain-size sensitive creep. The study could be supported by a brief consideration of other diffusion regimes.**

If we understand the reviewer's comment correctly, the reviewer suggests that grain-size sensitive (GSS) creep mechanisms other than diffusion creep may dominate. Although we do not exclude this possibility, it is difficult to evaluate it at the present stage because such mechanisms are poorly constrained for ringwoodite. For comparison, studies on olivine have suggested that, in addition to diffusion creep, dislocation-accommodated GSS, that is a non-linear GSS creep mechanism, may operate under certain conditions (e.g., Hirth and Kohlstedt, 2003; Ohuchi et al., 2015 *Sci.Adv.*). However, for ringwoodite, although microstructural evidence for GSS creep such as four grain junctions has been reported (Karato et al., 1998; ref9), no flow laws directly derived from deformation experiments have been established to date. Consequently. the diffusion-creep flow law constructed from Si diffusivity measurements by Shimojuku et al. (2009; ref17) is the only available framework that can be adopted in this study. We consider this approach robust because ringwoodite is extremely fine-grained, at least within NSBs, and dislocation-accommodated GSS creep is expected to operate primarily at coarser grain sizes, intermediate between diffusion and dislocation creep regimes.

- **Line 221: What is "stick-slip behavior"? The authors could introduce this concept to the**

reader.

A brief annotation (repetitive stress drops accompanied by transient slip events) has been added to the relevant section (line 216–217).

- **No mention is made of the effect of water. Water has a significant effect on deformation and grain growth. Is the mantle assumed to be totally dry? Did the authors attempt to measure the water contents in the samples used?**

The starting material used in our experiments contained 283±26 wt. ppm H_2O although we could not measure the water content of the recovered samples due to polishing and other procedures after embedding them in resin. The IR spectrum of the starting material exhibited a monomodal absorption band rather than the characteristic hydroxyl peaks within olivine crystals (3200–3600 cm^{-1}). This feature is interpreted to reflect the presence of small amounts of H_2O predominantly at grain boundaries (e.g., Long et al., 2011 *PEPI*; Demouchy et al., 2012 *PEPI*). We infer that the water content of the recovered samples is comparable to that of the starting material, as there is no significant source of water within our experimental cell assembly. This information is now briefly described in the Method section (lines 498-501).

Because the metastable olivine wedge is generally considered to be similarly dry, with water contents of a few hundred ppm or less (e.g., Kubo et al., 2009 *EPSL;* Kawakatsu and Yoshioka, 2011 *EPSL*), we conclude that there is no major issue regarding applicability to slab conditions of this study.

We agree that water has a significant influence on various rheological and kinetic processes under subduction zone conditions. In particular, deep earthquakes occurring outside the metastable olivine wedge may require water-related processes such as dehydration. However, these processes lie outside the scope of the present study and represent an important topic for future research.

- **Line 153: Was there any flow law determined from the experiments? No scaling law from laboratory to geophysical time scales is discussed. The authors should present a flow law from the experiments and discuss how the results can be projected from laboratory to geological timescales where typical strain rates are of the order of 10^{-15} s^{-1}.**

We did not determine a flow law from our experiments. Instead, we compared the measured olivine strength with that predicted by the flow law reported in the reference cited on line 151 (originally line 154) (Kawazoe et al., 2009; ref19). The observed olivine strength is consistent with the prediction of this flow law, as described in lines 149–151 (originally line 153).

We also described that this creep mechanism dominates the flow of metastable olivine at geological strain rates (~10^{-15} /s) (e.g., Liu and Zhang, 2015, ref. 32). (lines 292-294).

- **The phrase "On the other hand" is used repeatedly in the manuscript at the start of**

sentences/paragraphs.

We rephrased them as necessary (lines 147, 215, and 647).

- **Lines 244 – 248 and Lines 262 – 264: Two concepts are discussed by the authors, which can be a little confusing to the reader: Latent heat release due to phase transformation (reducing friction), and frictional heating. They suggest that latent heat can increase the temperature by 200 – 220 °C while frictional heating can increase in temperature by ~750 – 940 °C as a result of slip. The authors need to give clarity on the overall effect of temperature from these two concepts.**

Latent heat is generated at the onset of the phase transformation initiating slip, while frictional heat becomes significant during slip. The temperature increase due to frictional heat is shown in Fig. 4a, as described in line 255. Since localized deformation is considered to occur immediately following the onset of the transformation, we treat the effects of latent heat and frictional heating as effectively simultaneous and additive in terms of their contribution to temperature rise. Accordingly, we estimate the shear strain rate by assuming that both latent heat release and frictional heating collectively and locally increase the temperature (Fig. 4b and 4c). A minor revision has been made in the main text and figure caption to clarify these points (line 255 and lines 470-474).

Before the manuscript can be accepted for publication, I suggest that the above concerns be addressed.

We again thank the reviewer for the constructive comments. We have addressed all of the concerns raised, which have helped to improve the manuscript.

Reviewer #6 (Remarks to the Author):

I co-reviewed this manuscript with one of the reviewers who provided the listed reports. This is part of the Nature Communications initiative to facilitate training in peer review and to provide appropriate recognition for Early Career Researchers who co-review manuscripts.

We also sincerely appreciate the co-reviewer, in addition to Reviewer #5.
